# Statistical optimization of melanin-like pigment biosynthesis by marine Streptomyces isolate MOE3 and evaluation of its cytotoxic activity against A-431 cells

**DOI:** 10.1186/s12866-026-05590-8

**Published:** 2026-10-17

**Authors:** Moaz M. Hamed, Sahar W. M. Hassan, Eman H. Zaghloul

**Affiliations:** National Institute of Oceanography and Fisheries (NIOF), Cairo, Egypt

**Keywords:** Melanin-like pigment, Natural pigment, Marine actinomycetes, Marine biotechnology, Response surface methodology, Plackett–Burman design, Box–Behnken design

## Abstract

Marine actinomycetes represent a promising source of biologically active natural pigments. In the present study, isolate MOE3, recovered from mangrove sediment along the Egyptian Red Sea coast, was provisionally assigned to *Streptomyces lateritius* based on partial 16 S rRNA gene sequencing and phylogenetic analysis, showing approximately 99% sequence similarity to closely related Streptomyces taxa. The extracted pigment exhibited UV–Visible and FTIR characteristics consistent with a melanin-like pigment, including an absorption maximum at 255 nm. Plackett–Burman screening produced a maximum observed response approximately 1.65-fold relative to the basal response, while the highest experimentally observed response within the Box–Behnken design represented an approximately 2.3-fold increase relative to the basal response. The pigment also exhibited concentration-dependent in vitro cytotoxicity against A-431 human epidermoid carcinoma cells, with an IC₅₀ of 45.11 µg/mL after 24 h. These findings identify MOE3 as a promising microbial source of a melanin-like pigment and provide a basis for further structural, biological, and process-development studies.

**Graphical Abstract:**

**Figure:**
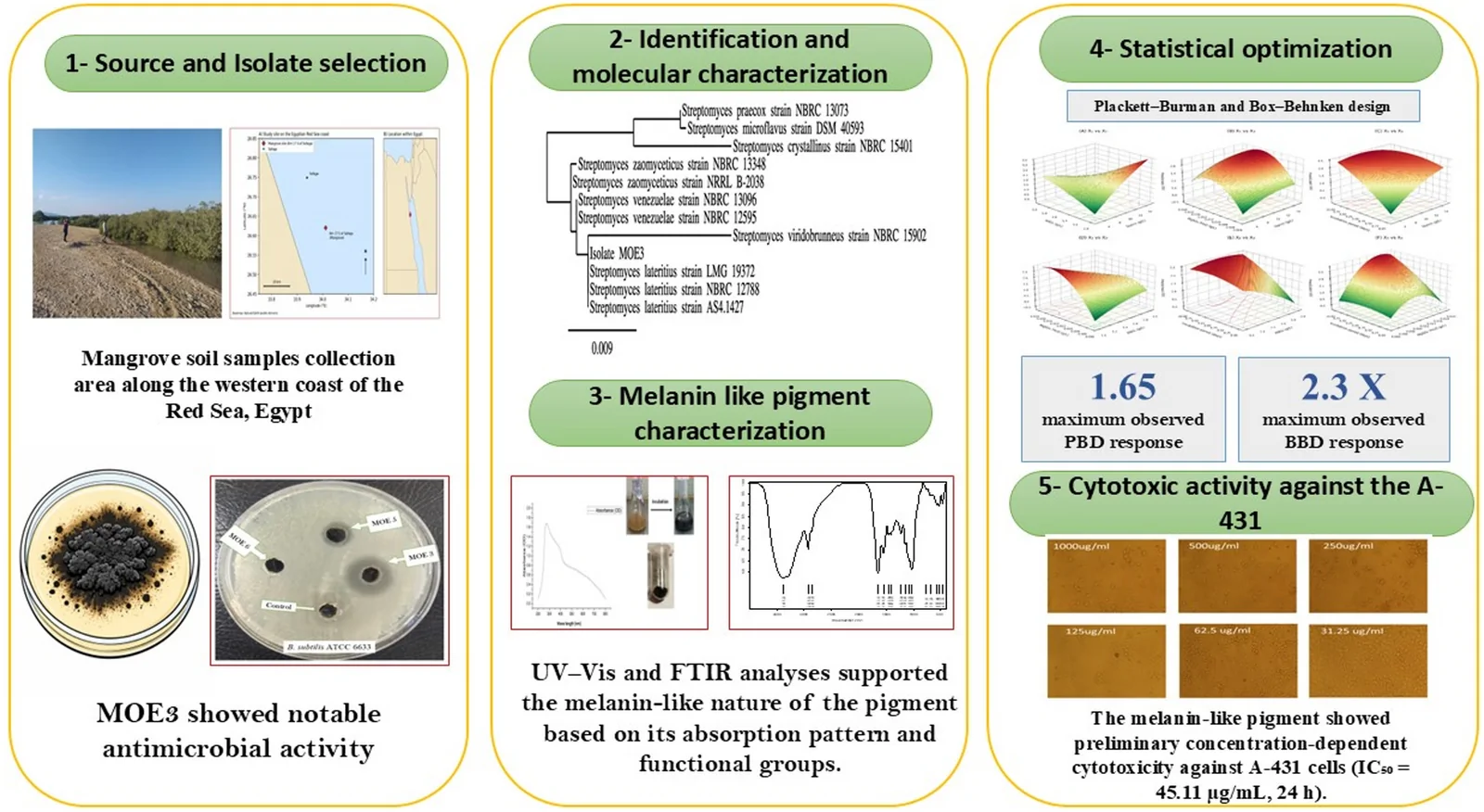

## Introduction

The use of biodegradable, non-toxic natural resources, product safety, and growing awareness of health and environmental concerns have attracted attention in several industrial sectors. Pigments are widely used in many industries, including food and medicine [1]. Compared to synthetic alternatives, natural pigments have attracted considerable attention due to their potential for positive biological effects, lower toxicity and carcinogenic potential, and environmental sustainability [2].

Actinomycetes, particularly those in the genus *Streptomyces*, are recognized as major producers of natural pigments [3]. These bacteria are Gram-positive and capable of synthesizing many pigments with beneficial biological qualities and colors, including actinorhodin, prodigiosin, and melanin [4]. Actinomycetes produce pigments that are in high demand for use in the food, cosmetics, textile, and pharmaceutical industries. These pigments have both functional and aesthetic benefits. Additionally, the sustainability of these microorganisms is a major benefit, since unlike synthetic pigments, which are often derived from petrochemicals, they can be grown on renewable substrates and are biodegradable [3].

The production of both natural and synthetic pigments significantly impacts the environment due to their potential to cause ecological harm. The market is increasingly transitioning to sustainable alternatives, such as natural pigments, primarily driven by regulatory constraints and rising consumer demand for eco-friendly goods. From this perspective, actinomycetes offer a promising source of natural, renewable, non-toxic, and biodegradable pigments that align with the increasing focus on sustainability across several industrial sectors [4].

Melanin, a heterogeneous biopolymer, has several roles in organisms. It is produced by oxidative polymerization using precursor molecules that are either phenolic or indolic [5, 6]. Based on its chemical structure, melanin is frequently categorized as eumelanin, pheomelanin, neuromelanin, pyomelanin, and allomelanin [7]. Nonetheless, because of its intricate polymerization properties and insoluble, amorphous nature, the chemical structure of melanin remains poorly characterized in the fields of biochemistry and biophysics. To tackle this challenge, researchers have used several complementary techniques to examine melanin structure [6]. Melanin’s distinct physicochemical properties make it useful in a wide range of applications. Melanin has several biological actions in the medical field, such as antibacterial, anticancer, anti-inflammatory, and antioxidant effects [5].

Additionally, melanins are widely used in the pharmaceutical and cosmetic fields because of their outstanding ability to absorb a wide range of electromagnetic radiation, such as visible light, ultraviolet light, and even X-rays [6]. From an environmental perspective, melanins play a significant role in bioremediation, particularly in the detoxification of contaminated sites, owing to their strong binding affinity for heavy metals and radionuclides [7]. Owing to these advantageous properties and their versatility across various applications, the demand for melanin production has increased substantially. Moreover, marine organisms represent a promising source of microbial melanin, attracting considerable interest from both academic research and industry, as they offer advantages such as safety, biodegradability, and environmental compatibility without causing harmful side effects [8].

Recent studies further emphasize the growing biotechnological interest in microbial and actinomycete-derived pigments. Streptomycetes have increasingly been recognized as promising microbial cell factories for melanin production because of their metabolic versatility and ability to produce extracellular pigments with potentially useful physicochemical and biological properties [9]. Recent advances have also highlighted actinomycete-derived pigments as promising natural colorants with potential applications in pharmaceutical, cosmetic, food, environmental, and material-related fields [4]. In 2026, an optimized *Streptomyces nigra* strain was reported to produce extracellular eumelanin at gram-per-liter levels, demonstrating the continuing development of Streptomyces-based biotechnological processes for microbial melanin production [10]. Furthermore, marine-derived *Streptomyces* strains continue to attract attention as sources of melanin and other bioactive pigments, as recently demonstrated for *Streptomyces* sp. SEA5 isolated from Persian Gulf sediments [11]. These recent findings reinforce the importance of exploring underinvestigated marine habitats as reservoirs of pigment-producing actinomycetes. Although melanin production has been reported in several *Streptomyces* species, available studies differ substantially from the present work in strain identity, ecological origin, and experimental scope. The marine-associated *Streptomyces cavourensis* SV21 was investigated primarily for the isolation and physicochemical comparison of soluble and insoluble melanin forms [12], whereas *Streptomyces djakartensis* NSS-3, recovered from terrestrial soil, was subjected to Plackett–Burman and Box–Behnken optimization and its melanin was evaluated against different cancer cell lines [13]. A marine-derived *Streptomyces* sp. UTMC 1334, closely related to *S. lateritius* based on 16 S rRNA gene analysis, was previously investigated for pyrrole-derived metabolites with anti-acetylcholinesterase activity rather than melanin production [14]. More recently, *Streptomyces* sp. SEA5 isolated from Persian Gulf marine sediment was reported as a marine source of eumelanin with antioxidant and potential pharmaceutical and skin-care applications [11]. Thus, the novelty of the present study does not reside in the statistical designs themselves, but in the integrated investigation of the Red Sea mangrove-derived isolate MOE3, provisionally assigned to *S. lateritius* as a melanin-like pigment producer, combining strain-specific production optimization, spectroscopic characterization, and biological evaluation. To the best of our knowledge, this specific integrated investigation has not previously been reported for a marine-derived isolate provisionally assigned to *S. lateritius*. This study aimed to isolate a melanin-producing marine actinomycete from mangrove sediment obtained from the Egyptian Red Sea coast, characterize the selected isolate and its pigment, statistically optimize pigment production using Plackett–Burman and Box–Behnken designs, and assess the cytotoxic activity of the extracted pigment against A-431 cells.

## Materials and methods

### Mangrove soil samples

**Fig. 1:**
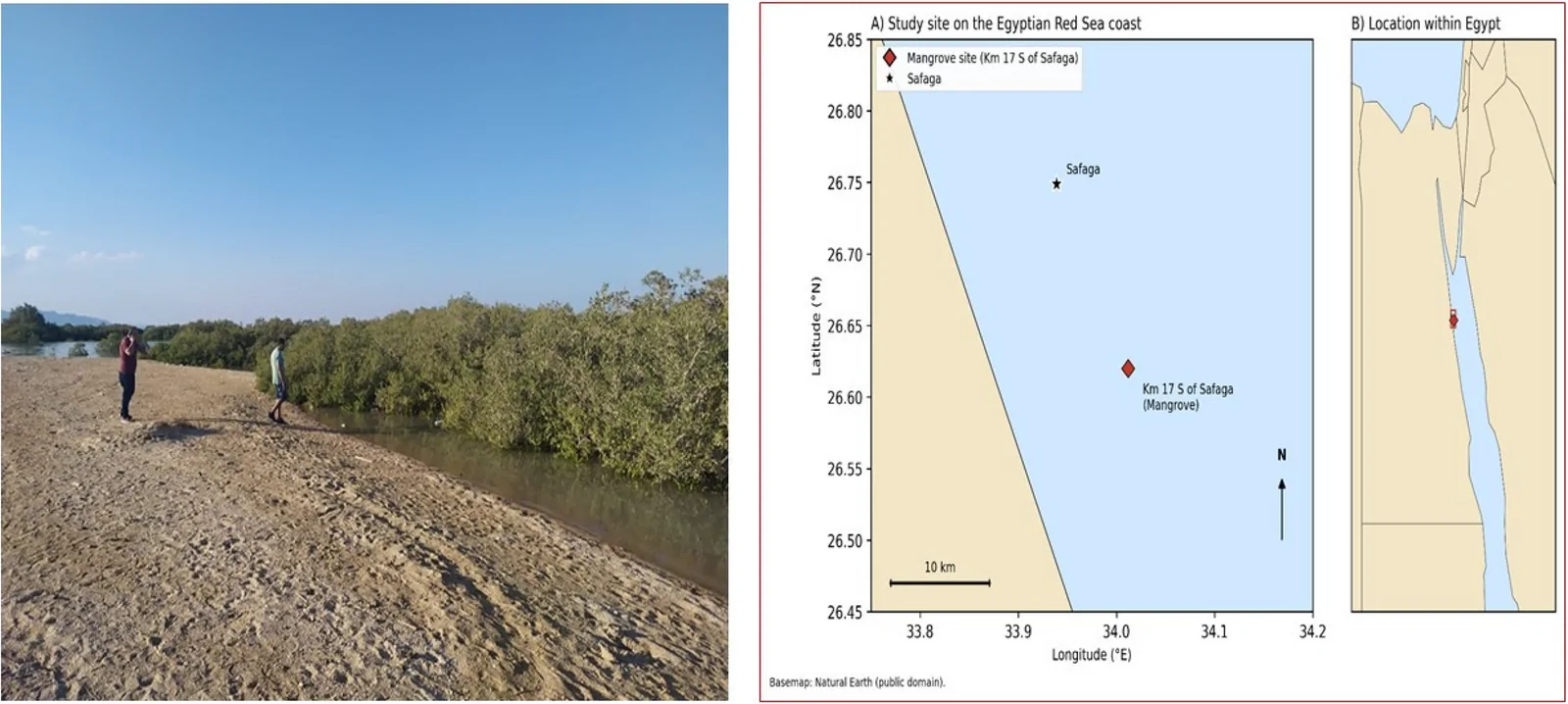
Mangrove soil samples collection area

Five sediment samples were collected from specific places throughout the mangrove environment in the summer of 2024. This mangrove area lies approximately 17 km south of Safaga City, Egypt, along the western coast of the Red Sea, between latitudes 26°36′53″N–26°37′07″N and longitudes 34°00′46″E–34°00′27″E (Fig. 1). Five surface sediment samples were obtained at a depth of 2–10 cm with a piston corer and were immediately placed into sterile plastic bags. The samples were then transferred to an ice box to the laboratory for bacteriological examination, which was consistently conducted within 24 h of collection.

### Isolation of actinomycetes

All sediment samples were pre-treated by air-drying at approximately 55 °C for 1 h. Starch casein agar (SCA) was employed as the isolation medium, consisting of (g/L): 10 g soluble starch, 0.3 g vitamin-free casein, 2 g KNO₃, 2 g NaCl, 2 g K₂HPO₄, 0.05 g MgSO₄·7 H₂O, 0.02 g CaCO₃, and 0.01 g FeSO₄·7 H₂O. The pH was adjusted to 7.0 ± 0.2, and 15 g of agar was incorporated [15]. Subsequently, all samples were subjected to serial dilution, and dilutions of 10⁻², 10⁻⁴, 10⁻⁶, and 10⁻⁸ were spread onto Petri plates containing solid SCA medium. Following inoculation, the plates were incubated at 32 ± 2 °C for approximately 15 days. After the incubation period, pigmented colonies were selected, purified, sub cultured, and preserved as stock cultures for further analyses. Actinomycetes were distinguished by their unique morphological characteristics, such as spore production, aerial mycelium, branched vegetative mycelium, and thick, leathery colonies. Colonies exhibiting these features were isolated from each sample by repeated transfers onto fresh SCA plates. This process continued until pure cultures were obtained, which was verified through examination of their morphological properties [16]. Pigmented colonies showing typical actinomycete morphology, including aerial mycelium, branched substrate mycelium, spore formation, and leathery colony texture, were repeatedly sub cultured on fresh SCA plates until morphologically homogeneous cultures were obtained. Culture purity was assessed by repeated examination of colony morphology during successive subculturing. Seven morphologically distinct actinomycete isolates were recovered, of which three (MOE3, MOE5, and MOE6) exhibited visible pigmentation and were selected for antibacterial screening.

### Screening for antibacterial activity

Seven actinomycete isolates were obtained; three of them were pigmented. The pigmented isolates MOE3, MOE5, and MOE6 were assessed for antibacterial activity using the agar well diffusion assay. The antibacterial spectrum was examined against four reference bacterial strains: two Gram-positive species, *Staphylococcus aureus* (ATCC 25923) and *Bacillus subtilis* (ATCC 6633), and two Gram-negative species, *Salmonella typhimurium* (ATCC 14028) and *Pseudomonas aeruginosa* (ATCC 9027). Bacterial inoculate were calibrated to a turbidity corresponding to the 0.5 McFarland standard to guarantee consistent cell density. To ensure uniform, confluent growth, sterile cotton swabs were used to inoculate freshly poured Mueller-Hinton agar (MHA) plates aseptically. After punching 8 mm-diameter wells into the inoculated agar, 100 µL of the cell-free supernatant was carefully added to each well. Sterile distilled water was included as the negative control. The plates were incubated at 37 °C for 24 h. After incubation, the clear zone diameter was measured in millimeters to determine the antibacterial ability. Each assay was performed in triplicate, and the inhibition-zone diameters were reported as mean ± SD [17].

### Identification and molecular characterization

Among the three pigmented isolates, MOE3 was selected for further investigation based on two predefined experimental criteria: its prominent black pigment production and its broader inhibition profile against the tested bacterial strains compared with MOE5 and MOE6. Genomic DNA was extracted from isolate MOE3 using the GeneJET Genomic DNA Purification Kit (Thermo Fisher Scientific, USA). Partial amplification of the 16 S rRNA gene was performed using DreamTaq Green PCR Master Mix (Thermo Fisher Scientific, USA) and the universal primer pair F-Primer (5′-AGAGTTTGATCCTGGCTCAG-3′) and R-Primer (5′-CGGTTACCTTGTTACGACTT-3′) [18]. The amplified PCR product was analyzed by electrophoresis on a 1% agarose gel stained with ethidium bromide and subsequently subjected to sequencing. The obtained partial 16 S rRNA gene sequence was compared with sequences available in the National Center for Biotechnology Information (NCBI) database using the Basic Local Alignment Search Tool (BLAST). The closest matches showed approximately 99% sequence similarity to members of the genus *Streptomyces*, with *Streptomyces lateritius* among the closest related taxa. Accordingly, isolate MOE3 was provisionally assigned to *S. lateritius* based on 16 S rRNA gene sequence similarity and phylogenetic analysis. The 16 S rRNA gene sequence of isolate MOE3 was deposited in the GenBank database under accession number PX944983. A phylogenetic tree was constructed using closely related reference sequences to evaluate the phylogenetic position of isolate MOE3.

### Pigment production, extraction, and quantification

A culture broth was prepared by inoculating a 250-mL flask containing 50 mL of Starch casein broth (SCB) medium with three freshly cut disks (9 mm diameter) of the selected isolate (MOE3), previously grown on SCA agar plates. The inoculated flasks were subsequently incubated at 30 °C for 7 days on a shaker incubator (200 rpm) as the baseline cultivation conditions for pigment production. Following incubation, the culture broth was centrifuged at 10,000 rpm for 20 min at 4 °C to remove cells and debris to obtain a clear solution before the black pigment was extracted. The resulting supernatant was carefully collected and subjected to acidification to pH 2 by using 6 M HCl for 4 h. Following that, the supernatant was centrifuged at 10,000 rpm for 20 min at 4 °C, and the precipitated pigment was collected. The obtained pellets were washed many times with distilled water and finally centrifuged at 10.000 rpm for 15 min [19]. All samples were protected from light by maintaining them under dark conditions to prevent pigment degradation. The extracted pigment was analyzed spectrophotometrically by scanning absorbance across the wavelength range of 200–800 nm. Quantification was performed according to Rudrappa et al. (2022) by recording the absorbance at 410 nm for comparative estimation of pigment production [19]. Pigment production was evaluated comparatively by spectrophotometric absorbance at 410 nm. Gravimetric recovery of the extracted pigment and absolute pigment yield expressed as g/L were not determined in the present study. The pigment-production response obtained under these baseline cultivation conditions was 1.7 OD at 410 nm, and this value was used as the basal reference for subsequent fold-change calculations.

### Spectroscopic characterization of the extracted pigment

#### UV–Vis spectrophotometer

To ascertain its spectral properties, the extracted pigment was dissolved in 1 N NaOH and scanned in the 200–800 nm range using a UV–Visible spectrophotometer (Model: 6800, JENWAY). The full scan was used to determine the characteristic absorption profile of the extracted pigment, whereas absorbance at 410 nm was used as a comparative spectrophotometric index of pigment production. Each sample was measured in triplicate, and the results were expressed as mean ± SD [6].

#### Fourier transform‑infrared (FT‑IR)

The Fourier Transform Infrared (FTIR) spectrum of the extracted pigment was recorded using an infrared spectrometer (PerkinElmer, USA). Measurements were carried out over the wavenumber range of 400–4000 cm⁻¹ using potassium bromide (KBr) pellets prepared from a homogeneous mixture of spectroscopic-grade KBr and the pigment sample, which was compressed under vacuum [20].

### Optimization of the pigment production

#### Statistical design for optimization of pigment production by marine *Streptomyces* isolate MOE3

Using the SCB medium, the Plackett–Burman experimental design [21] was applied to screen the effects of selected medium components and cultivation variables on pigment production by isolate MOE3. The Plackett-Burman design matrix (Table 1) had eight combinations of seven independent variables (Table 2). Run 9 represented the center-point condition, with all investigated variables set at their coded level 0. High (+ 1) and low (-1) values were evaluated for each component.

**Table 1:** The experimental findings for seven cultural variables using the Plackett-Burman design

Run	S	K	K2	Mg	pH	Temp	IP
1	-1	1	1	1	-1	-1	-1
2	1	1	-1	-1	-1	-1	1
3	1	-1	1	-1	-1	1	-1
4	-1	-1	-1	1	-1	1	1
5	1	-1	-1	1	1	-1	-1
6	-1	-1	1	-1	1	-1	1
7	-1	1	-1	-1	1	1	-1
8	1	1	1	1	1	1	1
9	0	0	0	0	0	0	0

**Table 2:** The degrees of independent factors influencing pigment synthesis

Factor	Symbol	Level		
		-1	0	1
Starch (g/L)	S	5	10	15
KNO_3_ (g/L)	K	1.5	2	2.5
K₂HPO₄ (g/L)	K_2_	1.5	2	2.5
MgSO₄·7 H₂O (g/L)	Mg	0.01	0.05	0.1
pH	pH	6	7	8
Temperature (℃)	T	30	32	35
Incubation period (D)	IP	5	7	9

* An inoculum volume of 1 mL from a 7-day-old culture (10⁵ CFU/mL) was added to each experimental flask

The effects of the seven independent factors on pigment production were assessed using the Plackett–Burman design. For each factor, the main effect was calculated as the difference between the mean response at the high level (+1) and the mean response at the low level (-1), as follows:

$$\begin{aligned}\mathrm{Main}\;\mathrm{effect}\;(\mathrm{Ei})\;=&\;\lbrack\mathrm{Sum}\;\mathrm{of}\;\mathrm{responses}\;\mathrm{at}\;\mathrm{the}\;\\&\mathrm{high}\;\mathrm{level}\;-\;\mathrm{Sum}\;\mathrm{of}\;\\&\mathrm{responses}\;\mathrm{at}\;\mathrm{the}\;\mathrm{low}\;\mathrm{level}\rbrack\;/\;\mathrm N\end{aligned}$$

where N represents the number of runs at each level. In the present eight-run Plackett–Burman design, *N* = 4.

The corresponding coded coefficient (βi) was calculated as half of the main effect:

$$\mathrm{Coded}\;\mathrm{coefficient}\;(\beta i)\;=\;\mathrm{Main}\;\mathrm{effect}\;(\mathrm{Ei})\;/\;2$$

Therefore:

$$\begin{aligned}\mathrm{Coded}\;\mathrm{coefficient}\;({\beta{i}})\;=&\;\lbrack\mathrm{Sum}\;\mathrm{of}\;\mathrm{responses}\;\mathrm{at}\;\mathrm{the}\;\\&\mathrm{high}\;\mathrm{level}\;-\;\mathrm{Sum}\;\mathrm{of}\;\\&\mathrm{responses}\;\mathrm{at}\;\mathrm{the}\;\mathrm{low}\;\mathrm{level}\rbrack\;\\&/\;(2\;\times\;\mathrm N)\end{aligned}$$

The Plackett–Burman design was used as an exploratory factor-screening approach. The estimated coded coefficients were used as descriptive screening measures to support the selection of variables for subsequent evaluation. No formal statistical ranking of the investigated factors was assigned. Because the eight-run, seven-factor design did not provide an independent residual-error estimate suitable for reliable formal inference, confidence intervals and exact p-values were not assigned to the screening coefficients.

#### Evaluation of culture conditions using the Box–Behnken design

Based on the Plackett–Burman screening results, four variables starch (X₁), KNO₃ (X₂), MgSO₄·7 H₂O (X₃), and incubation period (X₄) were selected for further evaluation using the Box–Behnken design (BBD) [22, 23]. The experimental levels investigated for these variables in the Box–Behnken design are presented in Table 7. Each variable was investigated at three coded levels (− 1, 0, and + 1), representing low, center, and high levels, respectively. A 27-run experimental design was used to evaluate the effects and interactions of these variables on pigment production. The Box–Behnken design comprised 27 experimental runs, and spectrophotometric measurements of pigment production were performed in triplicate. The experimental responses were fitted to a second-order polynomial model to describe the relationship between the selected variables and pigment production within the investigated experimental region. The relationship between the independent variables and pigment production was described using the following second-order polynomial equation:

$$\begin{aligned} Y &=\beta_0+\beta_1X_1+\beta_2X_2+\beta_3X_3\\ &\quad +\beta_4X_4+\beta_{12}X_1X_2+\beta_{13}X_1X_3\\ &\quad +\beta_{14}X_1X_4+\beta_{23}X_2X_3+\beta_{24}X_2X_4\\ &\quad +\beta_{34}X_3X_4+\beta_{11}X_1^2+\beta_{22}X_2^2\\ &\quad +\beta_{33}X_3^2+\beta_{44}X_4^2 \end{aligned}$$

Where Y represents the predicted response (pigment production); β₀ is the intercept; β₁–β₄ are the linear regression coefficients; β₁₂, β₁₃, β₁₄, β₂₃, β₂₄, and β₃₄ are the interaction coefficients; and β₁₁, β₂₂, β₃₃, and β₄₄ are the quadratic coefficients. X₁, X₂, X₃, and X₄ represent the coded values of starch, KNO₃, MgSO₄·7 H₂O, and incubation period, respectively. Model adequacy was evaluated collectively using R², adjusted R², predicted R², analysis of variance (ANOVA), lack-of-fit analysis, and residual diagnostics. Because the predicted R² was negative, the fitted quadratic model was interpreted primarily as a descriptive and exploratory model within the investigated experimental region rather than as a model with strong predictive capability (Table 3).

**Table 3:** The Box-Behnken experimental design for four factors

x1	x2	x3	x4	x1*x2	x1*x3	x1*x4	x2*x3	x2*x4	x3*x4	x1*x1	x2*x2	x3*x3	x4*x4
0	1	0	1	0	0	0	0	1	0	0	1	0	1
1	-1	0	0	-1	0	0	0	0	0	1	1	0	0
0	0	1	-1	0	0	0	0	0	-1	0	0	1	1
1	0	0	1	0	0	1	0	0	0	1	0	0	1
0	1	-1	0	0	0	0	-1	0	0	0	1	1	0
0	0	-1	1	0	0	0	0	0	-1	0	0	1	1
0	0	1	1	0	0	0	0	0	1	0	0	1	1
1	0	1	0	0	1	0	0	0	0	1	0	1	0
-1	0	1	0	0	-1	0	0	0	0	1	0	1	0
0	0	-1	-1	0	0	0	0	0	1	0	0	1	1
0	0	0	0	0	0	0	0	0	0	0	0	0	0
0	1	0	-1	0	0	0	0	-1	0	0	1	0	1
-1	0	0	1	0	0	-1	0	0	0	1	0	0	1
0	1	1	0	0	0	0	1	0	0	0	1	1	0
1	1	0	0	1	0	0	0	0	0	1	1	0	0
0	-1	0	-1	0	0	0	0	1	0	0	1	0	1
1	0	0	-1	0	0	-1	0	0	0	1	0	0	1
-1	0	-1	0	0	1	0	0	0	0	1	0	1	0
-1	-1	0	0	1	0	0	0	0	0	1	1	0	0
-1	0	0	-1	0	0	1	0	0	0	1	0	0	1
0	-1	-1	0	0	0	0	1	0	0	0	1	1	0
0	-1	1	0	0	0	0	-1	0	0	0	1	1	0
1	0	-1	0	0	-1	0	0	0	0	1	0	1	0
0	0	0	0	0	0	0	0	0	0	0	0	0	0
0	-1	0	1	0	0	0	0	-1	0	0	1	0	1
-1	1	0	0	−1	0	0	0	0	0	1	1	0	0
0	0	0	0	0	0	0	0	0	0	0	0	0	0

The factor effects identified during Plackett–Burman screening were interpreted within the concentration ranges tested in that design. Because the Box–Behnken design evaluated a distinct set of experimental factor ranges, the direction of the Plackett–Burman screening coefficients was not assumed to represent a direct directional prediction across the Box–Behnken design space.

#### Statistical analysis

The experimental data obtained from the Box–Behnken design were analyzed by multiple regression and fitted to a second-order polynomial model. The statistical significance of the regression coefficients was evaluated using Student’s t-test and the corresponding p-values. Exact two-sided p-values and 95% confidence intervals (95% CI) were reported for the estimated regression coefficients. Because the independent variables were expressed on a common coded scale (-1, 0, and + 1), the magnitude and direction of the regression coefficients were used to describe the relative effect of individual linear, interaction, and quadratic terms. Overall model effect and explanatory performance were summarized using R², adjusted R², and predicted R² rather than relying solely on statistical significance thresholds. Model adequacy was assessed using the coefficient of determination (R²), adjusted R², predicted R², analysis of variance (ANOVA), and lack-of-fit analysis. The predicted R² was calculated based on the prediction error sum of squares (PRESS) to evaluate the ability of the fitted model to predict observations excluded from model fitting. Residual diagnostics were also performed to assess the assumptions underlying the regression model. Studentized residuals were plotted against the predicted responses and experimental run order to examine possible systematic patterns, while a normal probability plot of the residuals was used to assess their approximate normal distribution. Lack of fit was evaluated relative to the pure experimental error estimated from the replicated center-point runs. Microsoft Excel 2025 was used for regression and statistical calculations, while STATISTICA 10.0 was used to generate response-surface plots.

### In vitro cytotoxicity assay against A-431 cells

The assessment of the extracted pigment cytotoxicity was performed against A-431 cells by inoculating 96-well tissue culture plates with 1 × 10⁵ cells/mL, followed by incubation at 37 °C for 24 h to allow the formation of a confluent monolayer. The human epidermoid carcinoma cell line (A-431) was obtained from Science Way for Scientific Research, Egypt, and used for the cytotoxicity assay. The growth medium was removed after formation of a confluent cell sheet, followed by two washes. The pigment sample was serially diluted in RPMI medium supplemented with 2% serum, which was used as the maintenance medium. Subsequently, 0.1 mL from each prepared dilution was added to separate wells. For each experiment, three wells containing untreated A-431 cells maintained in RPMI medium supplemented with 2% serum, without addition of the tested pigment, were included as the negative (untreated-cell) control. These control wells were used as the reference corresponding to 100% cell viability for calculation of the relative viability of pigment-treated cells. The plates were then incubated at 37 °C and monitored for any observable changes. An MTT solution was freshly prepared at a concentration of 5 mg/mL in PBS (Bio Basic Canada Inc.), and 20 µL of the MTT solution was added to each well. To ensure proper mixing, the plate was placed on a shaker at 150 rpm for 5 min, then incubated at 37 °C in a humidified atmosphere containing 5% CO₂ for 4 h to allow metabolic reduction of MTT. After incubation, the culture medium was carefully discarded, and the plates were left to dry to remove any remaining liquid. The resulting formazan crystals, produced through MTT metabolism, were dissolved by adding 200 µL of DMSO to each well. The plate was then shaken again at 150 rpm for 5 min to complete solubilization. The absorbance was recorded at 560 nm with background subtraction at 620 nm, and the corrected optical density was considered directly related to the number of viable cells [24]. Each tested concentration was evaluated in triplicate wells. Cell viability was calculated relative to the untreated control, which was considered as 100% viability. The IC₅₀ value was obtained from the laboratory dose–response analysis and is reported as mean ± SD.

## Results

### Isolation and screening

Bacterial isolation was done using sediment samples that were gathered from different parts of the mangrove forest. Using various cultivation techniques, three of the seven actinomycete isolates were determined to be pigmented actinomycetes. The pigmented isolates were screened for antibacterial activity using the agar well diffusion assay (Table 4). Compared to the other isolates, isolate MOE3 stood out for having the capacity to develop a distinctive black pigment therefore, it was chosen for this study’s more thorough examination.

**Table 4:** Assessment of the antibacterial potential of pigment-producing actinomycete isolates against bacterial pathogens

Bacterial pathogens	Inhibition zone diameter (mm)			Negative Control
	MOE3	MOE5	MOE6	D.W
*B. subtilis* ATCC 6633	16.0 ± 0.2	10.0 ± 0.2	0.0 ± 0.0	0.0 ± 0.0
*S. aureus* ATCC 25,923	16.0 ± 0.08	0.0 ± 0.0	0.0 ± 0.0	0.0 ± 0.0
*P. aeruginosa* ATCC 9027	20.0 ± 0.57	0.0 ± 0.0	0.0 ± 0.0	0.0 ± 0.0
*S. typhimurium* ATCC 14,028	18.0 ± 0.08	0.0 ± 0.0	0.0 ± 0.0	0.0 ± 0.0

Data are presented as mean ± SD of three independent measurements

**Fig. 2:**
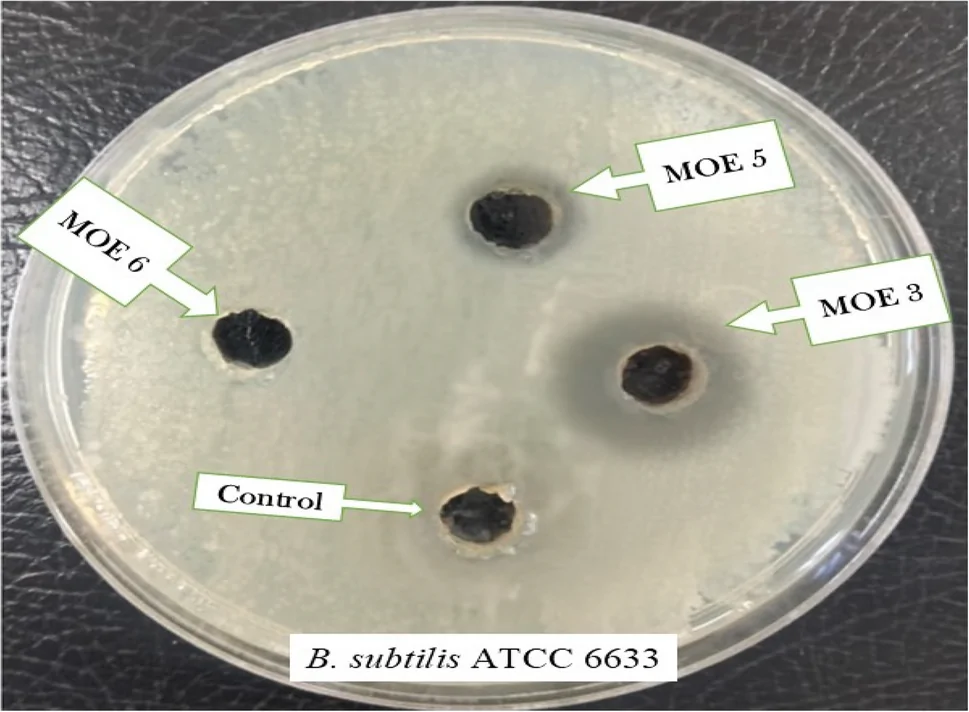
Inhibition zones of the pigmented actinomycete isolates MOE 3, MOE5, and MOE6 cell-free extracts against *Bacillus subtilis* ATCC 6633

Among the tested isolates, MOE3 produced inhibition zones against all four bacterial strains, ranging from 16.0 ± 0.08 to 20.0 ± 0.57 mm, whereas MOE5 showed detectable inhibition only against *B. subtilis* and MOE6 showed no detectable inhibition under the tested conditions (Fig. 2). These findings represent comparative agar-diffusion screening results and should not be interpreted as a quantitative determination of antibacterial potency.

### Identification and molecular characterization

**Fig. 3:**
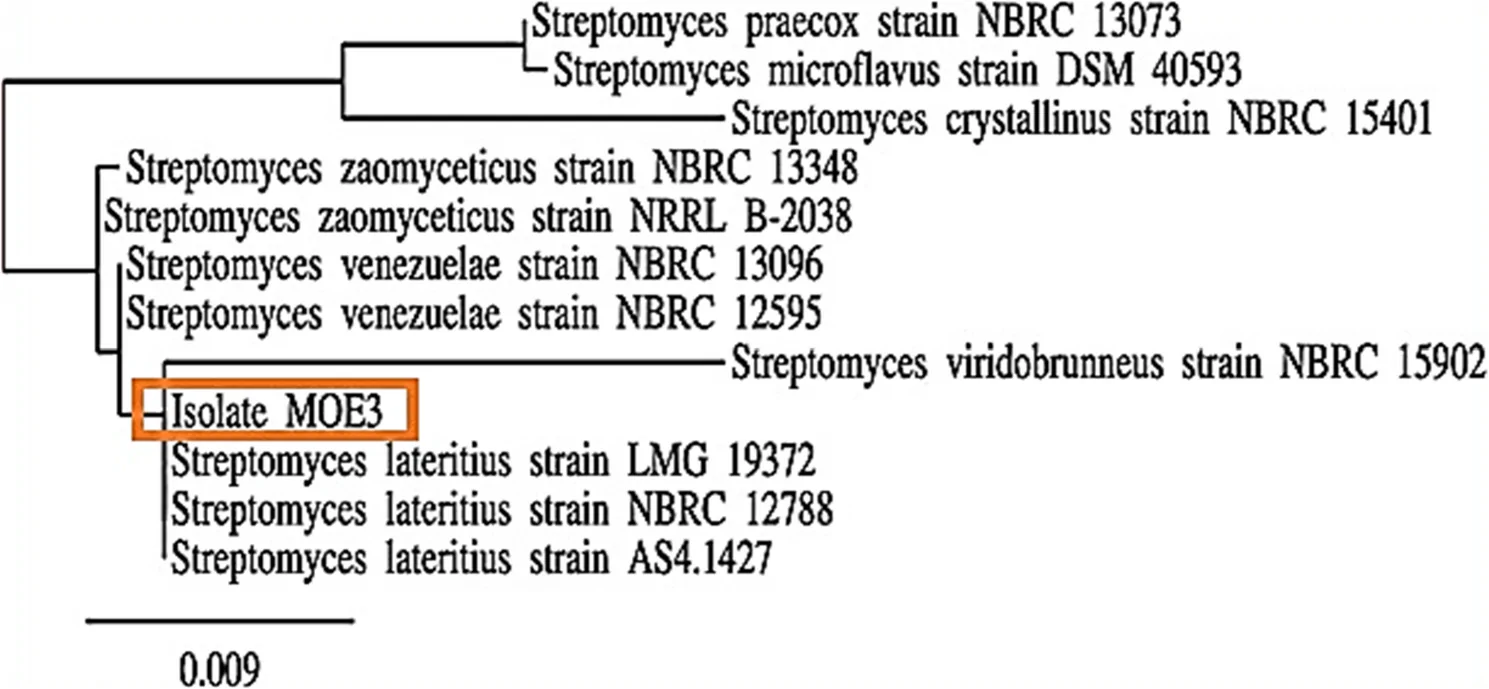
Phylogenetic tree based on the partial 16 S rRNA gene sequence of marine isolate MOE3, showing its close relationship to reference strains of *S. lateritius*

The marine actinomycete isolate MOE 3 was chosen for identification and molecular phylogenetic analysis based on the data obtained. After preparing genomic DNA, universal primers were used to partially amplify the 16 S rRNA gene. Agarose gel electrophoresis was used to identify the generated amplicons of the chosen isolate. BLAST analysis of the partial 16 S rRNA gene sequence showed approximately 99% sequence similarity to closely related members of the genus *Streptomyces*. Phylogenetic analysis placed isolate MOE3 in close association with reference strains assigned to *Streptomyces lateritius*. Based on these results, MOE3 was provisionally assigned to *S. lateritius*. The phylogenetic tree seen in Fig. 3 was created using the MOE 3 sequence, which was deposited in GenBank under accession number PX944983.

### UV absorption analysis

**Fig. 4:**
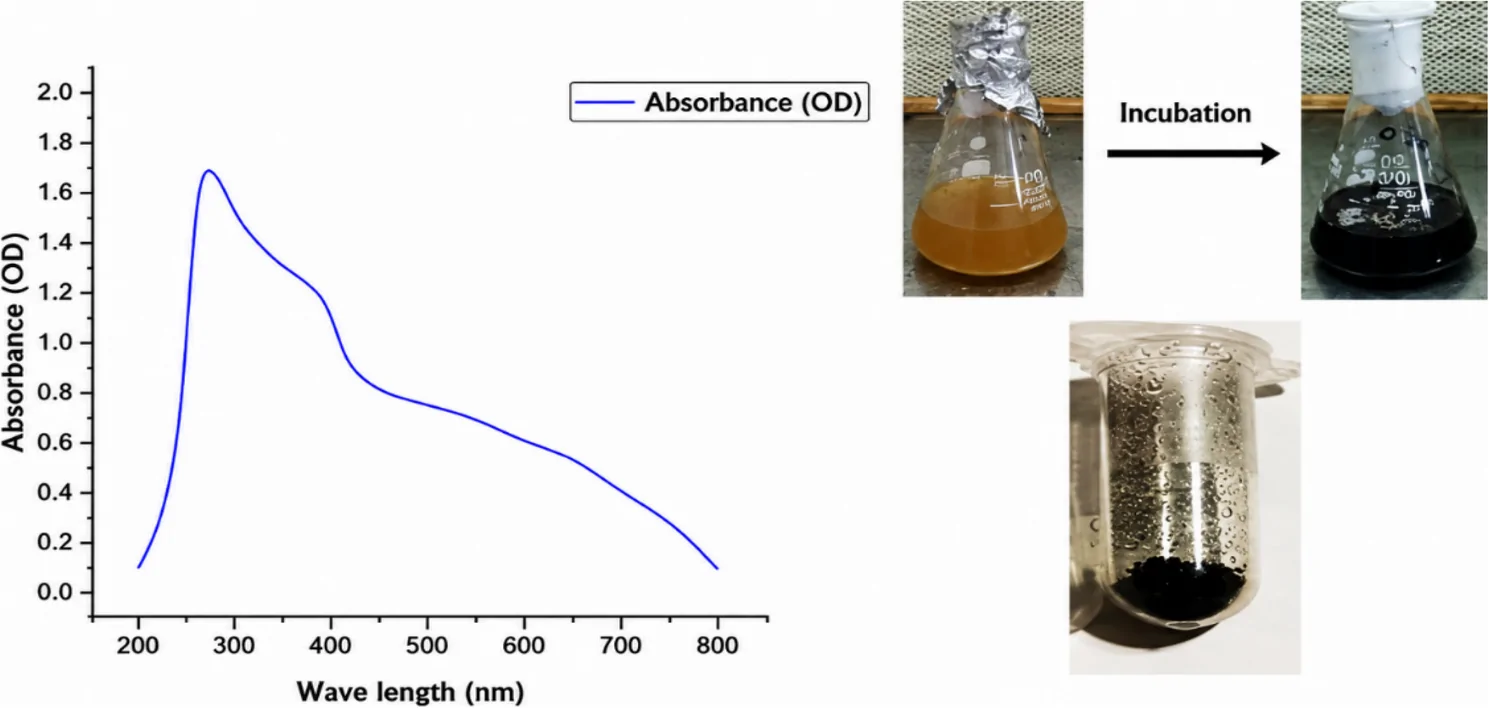
UV–Visible absorption spectrum of the pigment extracted from *Streptomyces sp.* MOE3, closely related to *S. lateritius*, showing a maximum absorption peak at 255 nm and a gradual decrease toward higher wavelengths

UV–Visible spectroscopic examination of the extracted pigment showed a distinct absorption maximum at 255 nm, after which the absorbance progressively declined as the wavelength increased. This spectral pattern is commonly associated with melanin-like pigments (Fig. 4).

### Statistical optimization of pigment production

To identify the key factors affecting black pigment biosynthesis by the marine actinomycete isolate MOE3, a Plackett–Burman experimental design was applied using the starch casein medium. The medium components selected for pigment production, along with culture-related parameters such as pH, incubation temperature, and incubation duration, were examined at two coded levels, low (−) and high (+), as listed in Table 2. The experimental responses obtained from the seven tested variables are provided in Table 5. The estimated coded screening coefficients were negative for KNO₃ and starch and positive for incubation period and MgSO₄·7 H₂O within the factor ranges investigated in the Plackett–Burman design. These four variables were subsequently selected for further evaluation using the Box–Behnken design; however, the signs of the screening coefficients were interpreted only within the Plackett–Burman ranges and were not assumed to predict directional behavior across the distinct Box–Behnken factor ranges (Table 6).

**Table 5:** Experimental design matrix and observed responses for pigment production using the Plackett–Burman design

Run	S	K	K2	Mg	pH	Temp	IP	Absorbance at 410 nm (OD)
1	-1	1	1	1	-1	-1	-1	1.5
2	1	1	-1	-1	-1	-1	1	0.5
3	1	-1	1	-1	-1	1	-1	1.2
4	-1	-1	-1	1	-1	1	1	2.5
5	1	-1	-1	1	1	-1	-1	1.6
6	-1	-1	1	-1	1	-1	1	2.8
7	-1	1	-1	-1	1	1	-1	0.6
8	1	1	1	1	1	1	1	0.8
9	0	0	0	0	0	0	0	1.7

The basal reference used for fold-change calculations (1.7 OD at 410 nm) was obtained under the baseline cultivation conditions described in Section "Pigment production, extraction, and quantification" (30 °C, 7 days) and was distinct from the Plackett–Burman center-point condition (Run 9)

**Table 6:** Estimated coded coefficient (β_i_) of the independent variables in the Plackett–Burman screening design

Variable	Estimated coded coefficient (β_i_)
Starch (g/L)	-0.413
KNO_3_ (g/L)	-0.588
K₂HPO₄ (g/L)	0.138
MgSO₄·7 H₂O (g/L)	0.163
pH	0.013
Temperature (℃)	-0.163
Incubation period (D)	0.213

The magnitude and direction of the estimated coded coefficients were used for factor screening. Formal inferential statistics were not assigned to the Plackett–Burman effects because the screening design did not provide an independent residual-error estimate suitable for reliable confidence intervals or exact significance testing

**Fig. 5:**
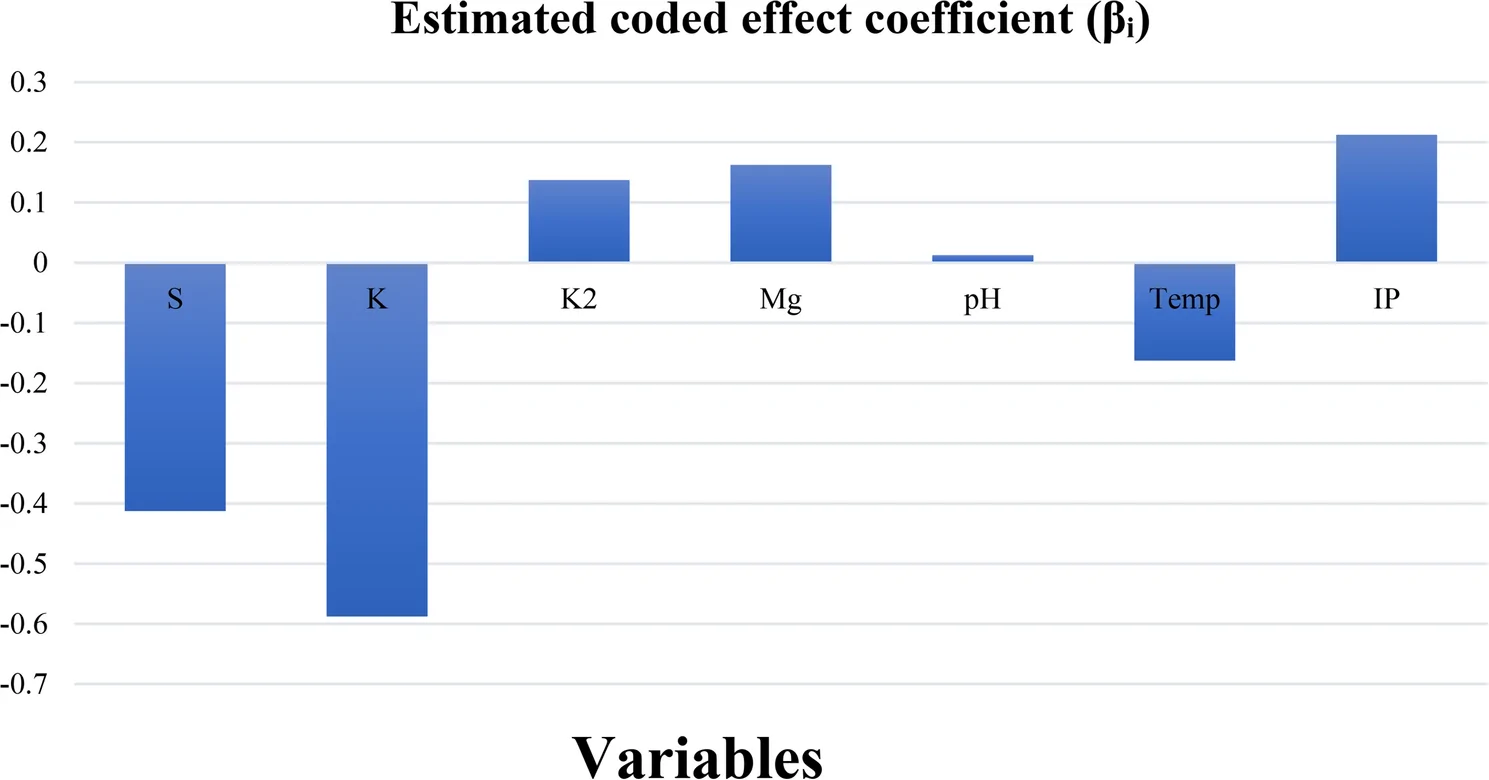
Elucidation of fermentation conditions affecting the pigment production by the marine actinomycete isolate MOE3

The primary impacts of the elements under investigation on absorbance were computed and shown in Fig. 5. According to these findings, the pigment formation was promoted by the positive (+) level of incubation duration and MgSO₄·7 H₂O, as well as the negative level (-) of starch and KNO_3_. These screening results were used to identify the culture variables taken forward for subsequent response-surface optimization.

Based on the exploratory Plackett–Burman screening results, starch, KNO₃, MgSO₄·7 H₂O, and incubation period were carried forward for subsequent evaluation using the Box–Behnken design. The screening coefficients were interpreted descriptively and were not used to establish a formal statistical ranking of the investigated factors.

### Response-surface evaluation of culture conditions using the Box–Behnken design

Based on the Plackett–Burman screening results, starch (X₁), KNO₃ (X₂), MgSO₄·7 H₂O (X₃), and incubation period (X₄) were selected for further evaluation using the Box–Behnken design. These factors were examined at three coded levels (− 1, 0, and + 1), as shown in Table 7, to evaluate their individual and interactive effects on pigment production within the investigated experimental region. Table 8 presents the experimental runs and their corresponding pigment-production responses. The experimental data were fitted to a quadratic polynomial model to describe the response surface within the investigated factor space.

**Table 7:** Experimental levels of the key independent variables selected for the Box–Behnken design to optimize pigment synthesis by isolate MOE3

Level	Starch (g/L)-X_1_	KNO3 (g/L)- X_2_	MgSO₄·7 H₂O (g/L)- X_3_	Incubation period(days)-X_4_
1	15	2	0.02	10
0	10	1.5	0.01	9
-1	5	1	0.005	8

**Table 8:** Box–Behnken design matrix showing the experimental runs and responses for pigment production by isolate MOE3

Ex	Starch X_1_	KNO_3_ X_2_	MgSO₄·7 H₂O X_3_	Incubation period X_4_	Absorbance at 410 nm (OD)
1	0	1	0	1	2.5
2	1	-1	0	0	2.3
3	0	0	1	-1	0
4	1	0	0	1	2.4
5	0	1	-1	0	0
6	0	0	-1	1	0
7	0	0	1	1	2.6
8	1	0	1	0	2
9	-1	0	1	0	0
10	0	0	-1	-1	0
11	0	0	0	0	2.2
12	0	1	0	-1	0
13	-1	0	0	1	2
14	0	1	1	0	0
15	1	1	0	0	0
16	0	-1	0	-1	2.2
17	1	0	0	-1	2.5
18	-1	0	-1	0	0
19	-1	-1	0	0	0
20	-1	0	0	-1	0
21	0	-1	-1	0	0
22	0	-1	1	0	3.9
23	1	0	-1	0	1.8
24	0	0	0	0	1.6
25	0	-1	0	1	2.8
26	-1	1	0	0	3.5
27	0	0	0	0	2.6

$$\begin{aligned}\mathrm Y\;=&\;2.133\;+\;\;0.458{\mathrm X}_1\;-\;0.433{\mathrm X}_2\;\\&+\;0.558{\mathrm X}_3\;+\;0.633{\mathrm X}_4\;-\;1.45{\mathrm X}_1{\mathrm X}_2\;\\&+\;0.05{\mathrm X}_1{\mathrm X}_3\;-\;0.525{\mathrm X}_1{\mathrm X}_4\;-\;0.975{\mathrm X}_2{\mathrm X}_3\;\\&+\;0.475{\mathrm X}_2{\mathrm X}_4\;+\;0.65{\mathrm X}_3{\mathrm X}_4\;-\;0.275{\mathrm X}_1^2\;\\&-\;0.187{\mathrm X}_2^2\;-\;1.05{\mathrm X}_3^2\;-\;0.212\mathrm X_{4}^{2}\end{aligned}$$

The regression coefficients are presented in Table 9, whereas the analysis of variance (ANOVA) and lack-of-fit results are summarized in Table 10.

**Table 9:** Regression coefficients, 95% confidence intervals, t-values, and exact p-values for the Box–Behnken quadratic model of melanin-like pigment production

Model term	Coefficient	95% CI	t-value	Exact *p*-value
Intercept (β₀)	2.133	0.981 to 3.286	4.033	0.0017
X₁ Starch	0.458	-0.118 to 1.035	1.733	0.1087
X₂ KNO₃	-0.433	-1.010 to 0.143	-1.638	0.1273
X₃ MgSO₄·7 H₂O	0.558	-0.018 to 1.135	2.111	0.0564
X₄ Incubation period	0.633	0.057 to 1.210	2.394	0.0339
X₁X₂	-1.450	-2.448 to -0.452	-3.165	0.0081
X₁X₃	0.050	-0.948 to 1.048	0.109	0.9149
X₁X₄	-0.525	-1.523 to 0.473	-1.146	0.2741
X₂X₃	-0.975	-1.973 to 0.023	-2.128	0.0547
X₂X₄	0.475	-0.523 to 1.473	1.037	0.3203
X₃X₄	0.650	-0.348 to 1.648	1.419	0.1814
X₁²	-0.275	-1.139 to 0.589	-0.693	0.5014
X₂²	-0.188	-1.052 to 0.677	-0.473	0.6450
X₃²	-1.050	-1.914 to -0.186	-2.647	0.0213
X₄²	-0.213	-1.077 to 0.652	-0.536	0.6020

**Table 10:** Analysis of variance and lack-of-fit assessment for the Box–Behnken quadratic model

Source	Sum of squares	df	Mean square	F-value	*p*-value
Model	35.3458	14	2.5247	3.0073	0.0316
Residual error	10.0742	12	0.8395	—	—
Lack of fit	9.5675	10	0.9568	3.7766	0.2274
Pure error	0.5067	2	0.2533	—	—
Total	45.4200	26	—	—	—

**Fig. 6:**
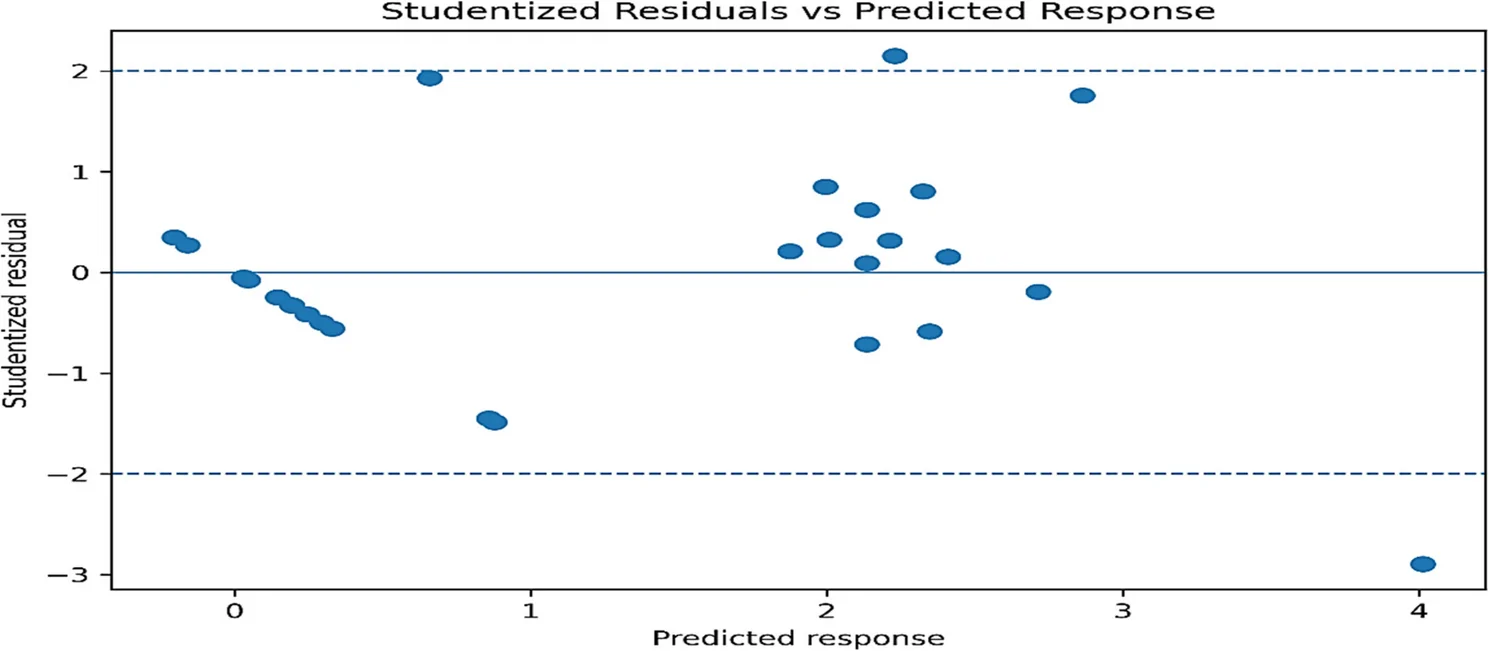
Studentized residuals versus predicted responses for the fitted box–Behnken quadratic model of melanin-like pigment production

**Fig. 7:**
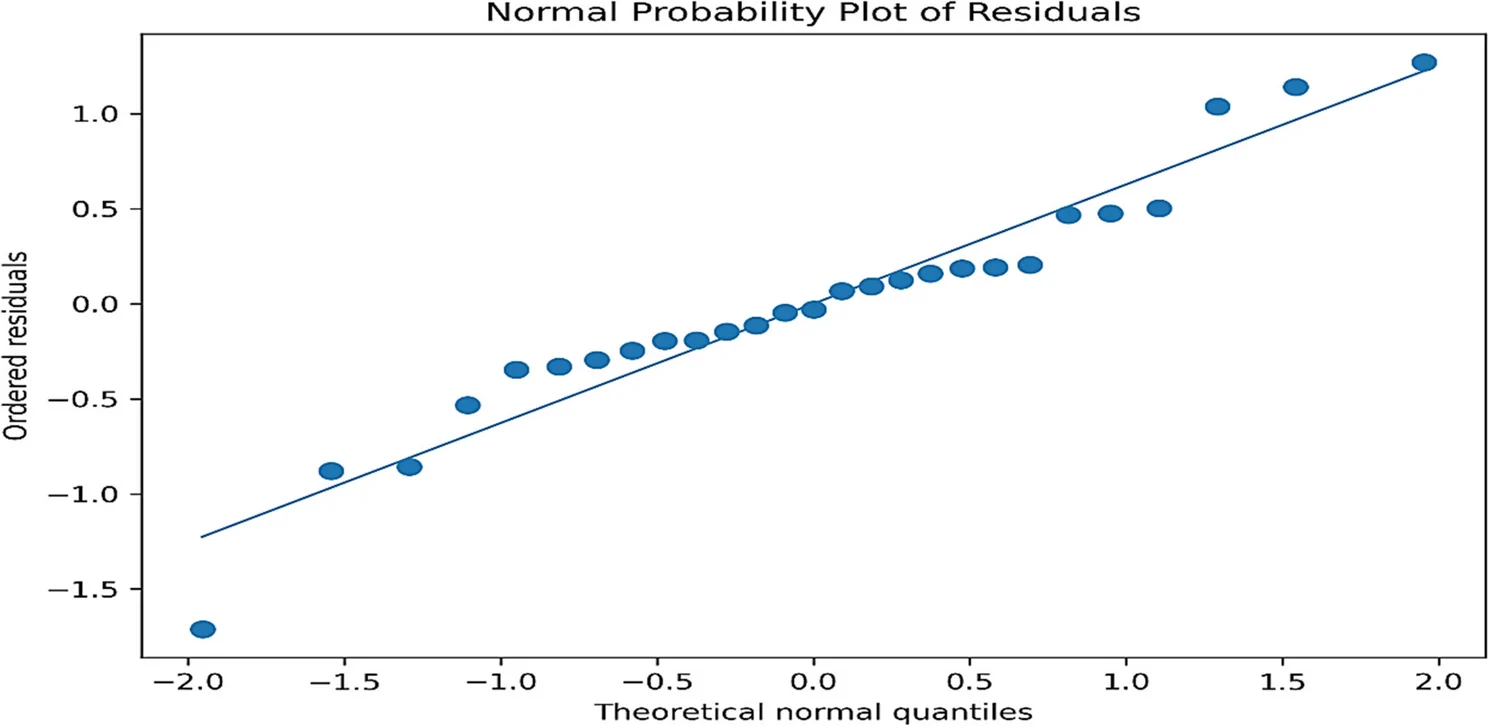
Normal probability plot of residuals obtained from the fitted box–Behnken quadratic model

**Fig. 8:**
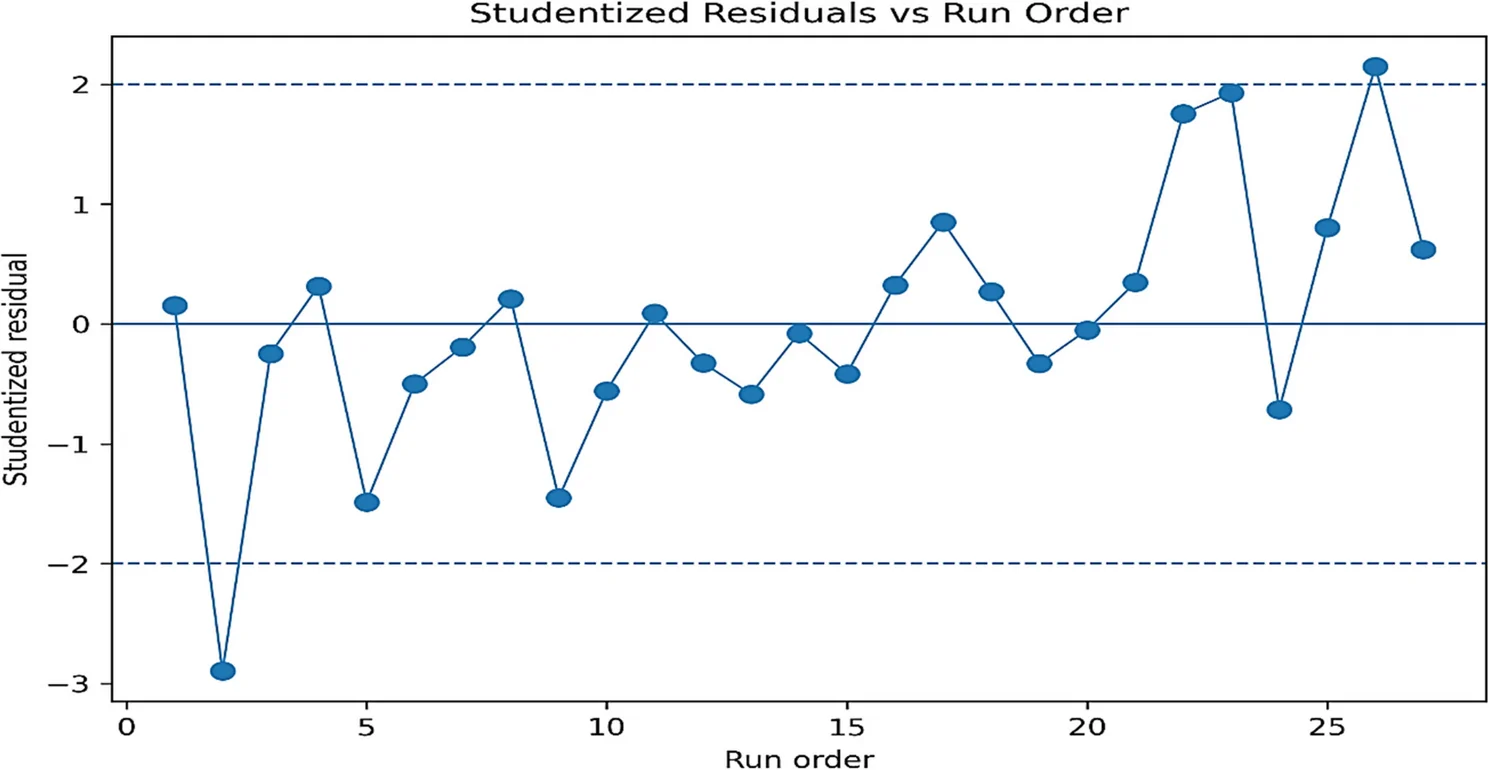
Studentized residuals versus experimental run order for the fitted box–Behnken quadratic model

**Fig. 9:**
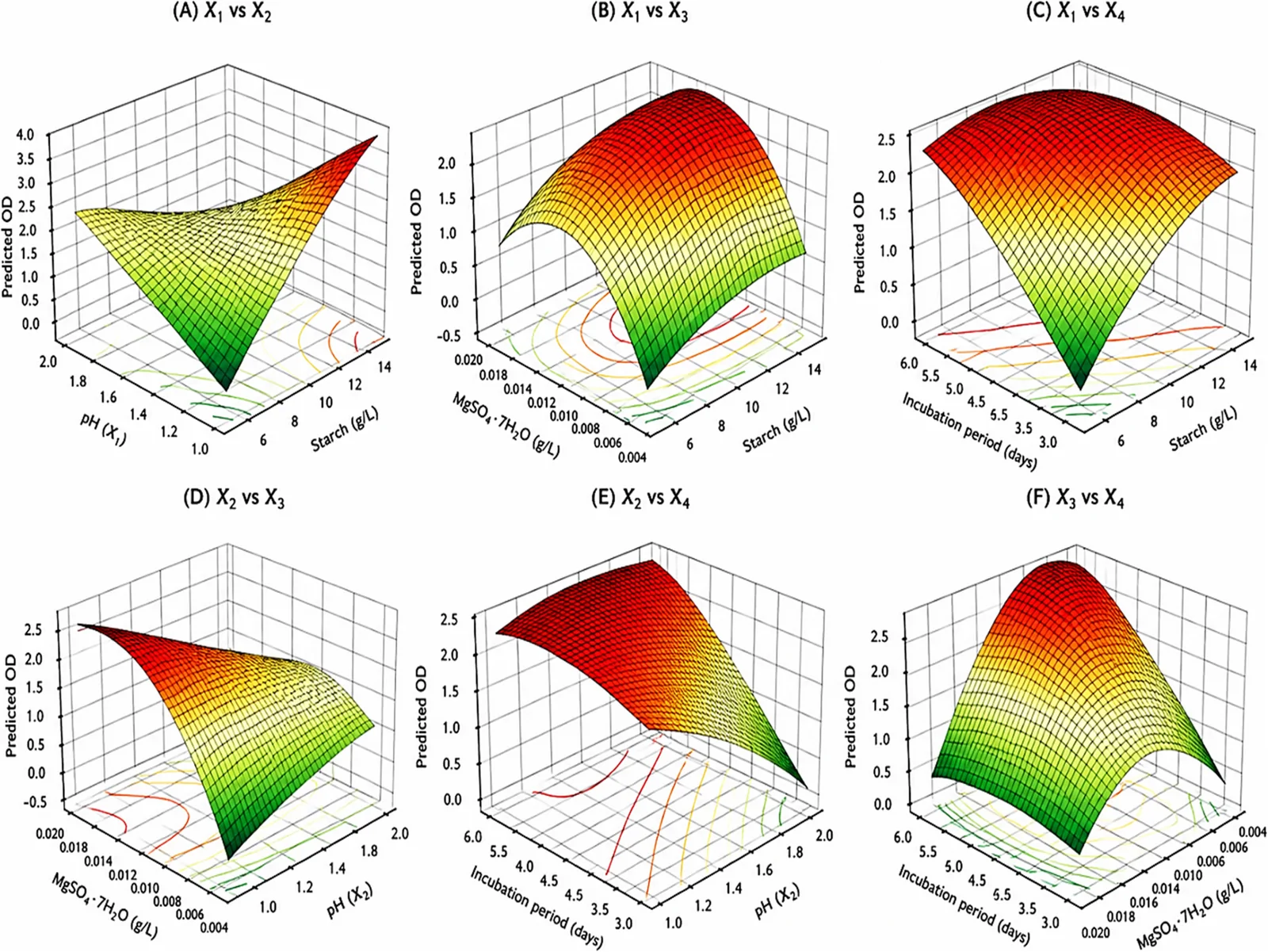
Three-dimensional response surface plots illustrating the estimated interaction effects of the four selected variables on melanin-like pigment production by isolate MOE3 based on the fitted Box–Behnken quadratic model: (**A**) starch and KNO₃, (**B**) starch and MgSO₄·7 H₂O, (**C**) starch and incubation period, (**D**) KNO₃ and MgSO₄·7 H₂O, (**E**) KNO₃ and incubation period, and (**F**) MgSO₄·7 H₂O and incubation period. In each plot, the remaining two variables were maintained at their center levels

The adequacy of the fitted box–Behnken quadratic model was assessed using several complementary statistical criteria. The model was statistically significant overall (F = 3.007, *p* = 0.0316), indicating that the fitted regression explained a significant proportion of the variation in pigment production within the investigated experimental region. The coefficient of determination (R²) was 0.7782, indicating that 77.82% of the observed response variability was explained by the model, whereas the adjusted R² was 0.5194. The regression coefficients and their 95% confidence intervals were additionally considered to evaluate the magnitude and precision of the estimated effects. Among the individual terms, the X₁X₂ interaction showed the largest absolute coefficient (β = −1.450, 95% CI: −2.448 to − 0.452, *p* = 0.0081), followed by the quadratic effect of MgSO₄·7 H₂O (X₃²; β = −1.050, 95% CI: −1.914 to − 0.186, *p* = 0.0213). The incubation-period linear effect was also statistically supported (β = 0.633, 95% CI: 0.057–1.210, *p* = 0.0339). These estimates provide information on effect magnitude and precision in addition to statistical significance. Lack-of-fit analysis was not statistically significant (F = 3.777, *p* = 0.227), suggesting that no significant systematic lack of fit could be detected relative to the pure experimental error within the studied design region. Nevertheless, the predicted R² value was negative (-0.2384), indicating poor cross-validated predictive performance for observations omitted from model fitting. Therefore, the model should not be interpreted as having strong general predictive ability. Rather, its utility is primarily descriptive and exploratory within the investigated factor space. Residual diagnostic plots (Figs. 6, 7 and 8) were additionally examined to evaluate model assumptions. The normal probability plot of residuals (Fig. 7) showed an approximately linear distribution, while the studentized residuals versus predicted responses (Fig. 6) and versus experimental run order (Fig. 8) showed no obvious systematic trend, although several observations exhibited relatively large residual deviations. Within the Box–Behnken experimental runs, the highest observed response was 3.9 OD at 410 nm (Run 22), corresponding to approximately a 2.3-fold increase relative to the basal response of 1.7 OD. Because the fitted quadratic model showed poor cross-validated predictive performance, as indicated by the negative predicted R², no claim of a generally predictive optimum was made. The model was therefore interpreted primarily as a descriptive and exploratory tool for examining factor effects and interactions within the investigated experimental region. Interaction effects of the four selected variables on pigment production were further illustrated using three-dimensional response surface plots generated from the Box–Behnken model using STATISTICA 10.0 software (Fig. 9). The response-surface plots illustrate the fitted relationships among starch concentration (X₁), KNO₃ (X₂), MgSO₄·7 H₂O (X₃), incubation period (X₄), and the predicted pigment-production response within the investigated factor space. Because several model terms were not statistically significant and the fitted model showed poor cross-validated predictive performance, the response surfaces were interpreted descriptively rather than as evidence of a reliable predictive optimum. Among the fitted terms, the starch–KNO₃ interaction (X₁X₂), the linear effect of incubation period (X₄), and the quadratic effect of MgSO₄·7 H₂O (X₃²) were statistically significant. Therefore, the response surfaces were used primarily to visualize local factor relationships and interactions within the tested experimental ranges.

### Fourier transform‑infrared (FT‑IR) spectrum analysis

**Fig. 10:**
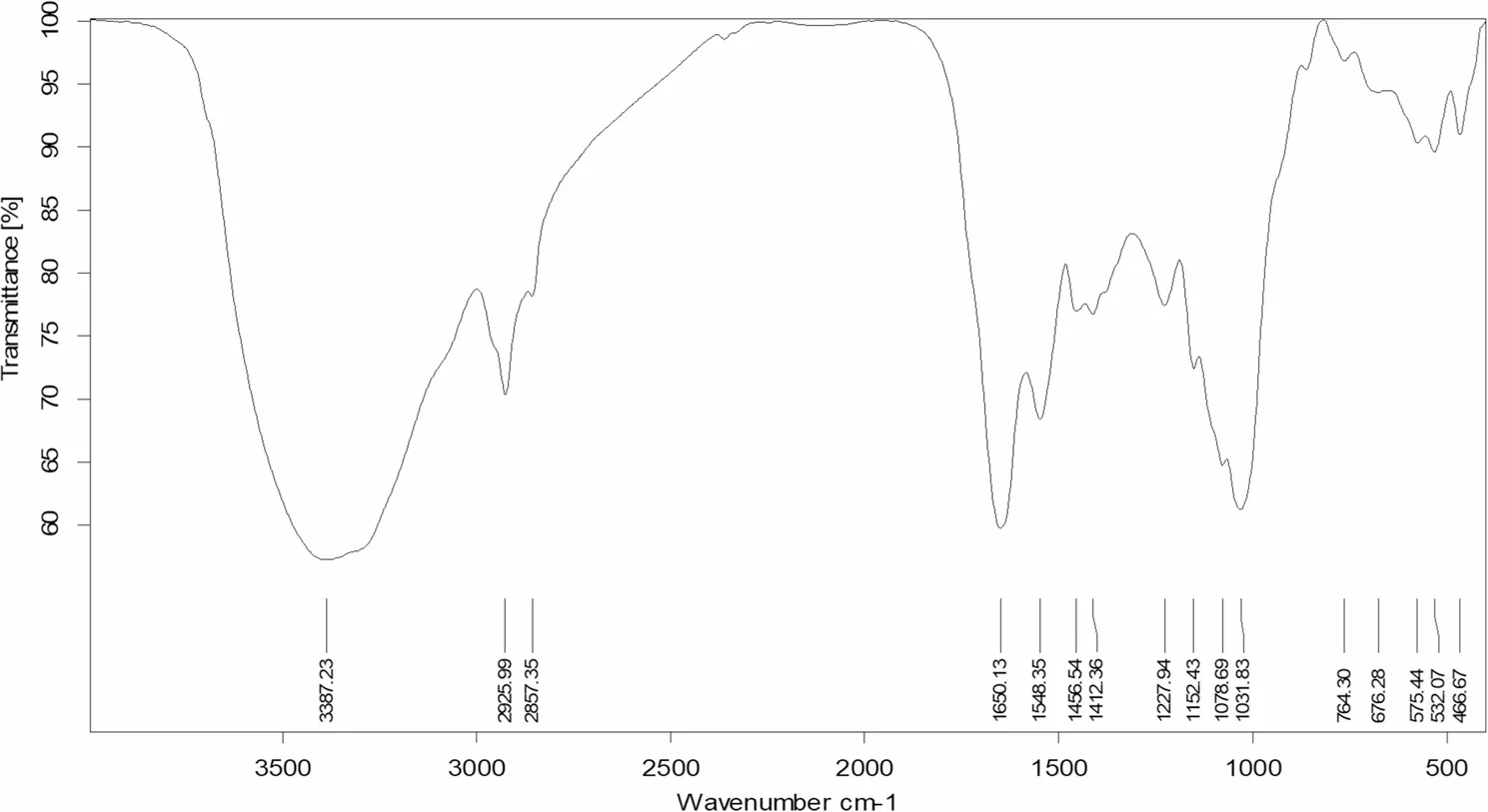
FTIR spectrum of the MOE3 extracted pigment

The FT-IR spectrum of the extracted pigment showed several absorption bands indicative of different functional groups (Fig. 10). The broad band at approximately 3387 cm⁻¹ was consistent with O–H stretching, while the bands at approximately 2926 and 2857 cm⁻¹ were attributed to aliphatic C–H stretching vibrations. Bands at approximately 1650 and 1548 cm⁻¹ may be associated with aromatic/conjugated and amide-related vibrations. The bands at approximately 1457 and 1412 cm⁻¹ may be attributed to C–H bending vibrations, while bands at approximately 1228 and 1152 cm⁻¹ may reflect C–O and/or C–N stretching vibrations. The band at 1078.69 cm⁻¹ may be associated with C–O and/or C–N stretching vibrations. The band at 764.30 cm⁻¹ may be associated with aromatic C–H out-of-plane bending vibrations. Overall, these spectral features are broadly consistent with reported melanin-like pigments but are not sufficient for definitive structural assignment.

### Cytotoxic activity against A-431 cells

**Fig. 11:**
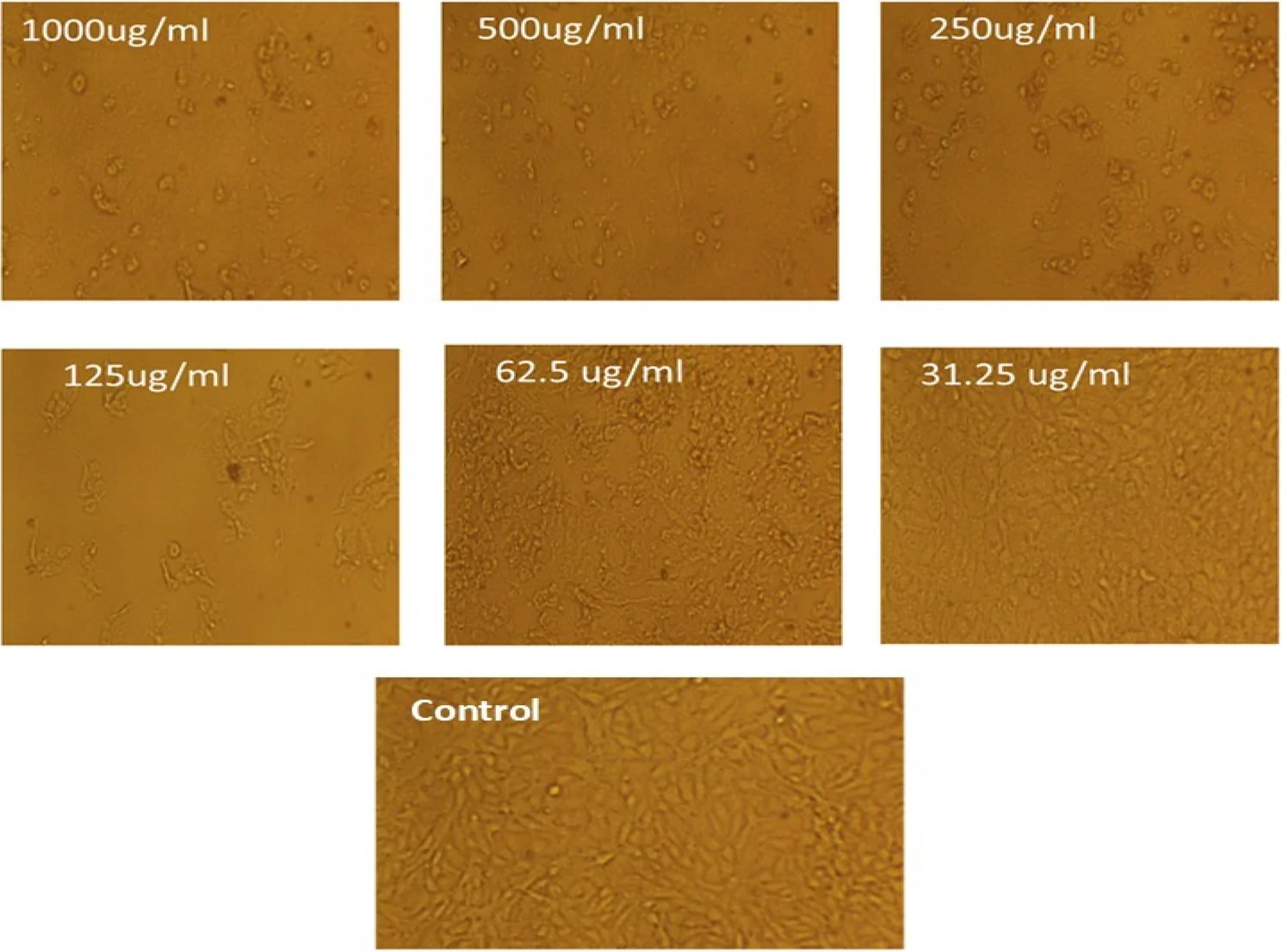
Photographs illustrating the effect of different concentrations of the extracted pigment on the growth of the A-431 cell line compared to control

The in vitro cytotoxic activity of the extracted melanin-like pigment was evaluated against the human epidermoid carcinoma cell line A-431 over a concentration range of 31.25–1000 µg/mL using the MTT assay. Untreated A-431 control cells maintained in the maintenance medium showed a mean optical density of 0.721 and were defined as 100% viable (0% cytotoxicity). Cell viability at each pigment concentration was expressed relative to this untreated control. As shown in Table 11, treatment with the pigment resulted in a concentration-dependent reduction in A-431 cell viability after 24 h of exposure. Cell viability decreased from 64.49% at 31.25 µg/mL to 2.91% at the highest tested concentration of 1000 µg/mL. The calculated IC₅₀ value was 45.11 ± 0.85 µg/mL. Microscopic examination also revealed marked morphological alterations in treated A-431 cells at higher pigment concentrations, as illustrated in Fig. 11. These findings demonstrate an in vitro cytotoxic effect of the extracted pigment against A-431 cells under the experimental conditions used.

**Table 11:** Viability (%) of A-431 cells after exposure to different concentrations

Pigment concentration (µg/mL)	Cell viability (%)	Cell toxicity (%)	IC_50_ ± SD
1000	2.91	97.08	45.11 ± 0.85
500	3.00	96.99	
250	3.37	96.62	
125	3.65	96.34	
62.5	22.88	77.11	
31.25	64.49	35.50	
(Untreated control) 0	100	0	

## Discussion

Actinomycetes are significant bacteria in biotechnology that synthesize different pigments for industrial use. Numerous promising pharmaceutically significant chemicals can be found in the marine environment, making it an intriguing research field [25]. Marine actinomycetes exhibit remarkable productivity and yield interesting compounds in comparison to their terrestrial counterparts. Marine actinomycetes inhabit not just ocean water and sediment but also many marine ecosystems, including estuary environments, seagrasses, fish, phytoplankton, mangroves, and coral reefs. Although marine actinomycetes have attracted increasing attention as producers of bioactive secondary metabolites, their full potential remains insufficiently explored. Among microorganisms, actinomycetes are particularly notable for synthesizing a broad spectrum of pigments under different culture conditions, whether on natural or artificial media. Compared with other pigment sources, microbial production is advantageous due to fast biomass development and the ability to utilize inexpensive substrates. Accordingly, actinomycete-derived pigments are gaining interest as a developing research area with wide-ranging industrial relevance [26].

The present study demonstrates the isolation, identification, and statistical optimization of melanin-like pigment produced by a marine actinomycete isolate obtained from the mangrove ecosystem of the Egyptian Red Sea coast. Marine environments, particularly mangrove sediment, are increasingly recognized as reservoirs of metabolically versatile actinomycetes capable of producing structurally diverse bioactive compounds [27]. In this context, the isolation of strain MOE3, provisionally assigned to *Streptomyces lateritius* highlights the biotechnological potential of underexplored marine niches to produce natural pigments.

Three of the seven recovered actinomycete isolates were recognized as pigmented actinomycetes using various cultivation techniques. Among these isolates, MOE3 stood out due to its distinctive phenotypic traits, specifically its capacity to create a recognizable black pigment, which set it apart from the other isolates and supported its selection for additional in-depth analysis. Among the three pigmented isolates, MOE3 showed the broadest inhibition profile in the agar well diffusion screening assay, producing measurable inhibition zones against both Gram-positive (*S. aureus* and *B. subtilis*) and Gram-negative (*S. typhimurium* and *P. aeruginosa*) test strains. The largest inhibition zone was observed against *P. aeruginosa* (20.0 ± 0.57 mm), while inhibition zones of 16.0–18.0 mm were recorded against the remaining tested bacteria. In contrast, MOE5 showed detectable inhibition only against *B. subtilis*, whereas MOE6 showed no detectable activity under the tested conditions. These findings supported the selection of MOE3 for further investigation. However, the antibacterial assay performed in the present study was intended as an initial screening procedure based on inhibition-zone measurements. MIC and MBC values were not determined, and the activity of the cell-free supernatant was not compared directly with standard antibiotics. Consequently, the present results do not permit quantitative assessment of antibacterial potency, bacteriostatic versus bactericidal effects, or comparison with clinically established antimicrobial agents. Therefore, the antibacterial findings should be considered preliminary screening evidence. Future studies should determine MIC and MBC values, include appropriate reference antibiotics as positive controls, and perform statistically supported comparative analyses to more rigorously evaluate the antibacterial activity associated with MOE3. Melanins, such as pyomelanin (dark brown), produced by a variety of Streptomyces, including e.g., *Streptomyces fuscus*, *Nocardiopsis* sp., and *Actinoalloteichus cyanogriseus*, have cytotoxic, antioxidant, antibacterial, drug-binding, and anticancer effects [13, 19, 28–30]. The diverse bioactive properties of pyomelanin make it a promising candidate for several applied fields, including cancer-related therapeutics, antimicrobial agents, and anti-aging cosmetics. In particular, its ability to protect against UV-induced damage and oxidative stress supports its potential value in pharmaceutical and cosmetic formulations [4].

The partial 16 S rRNA gene sequence placed MOE3 within the genus *Streptomyces* and showed close relatedness to reference strains of *S. lateritius*. These findings support the provisional assignment of MOE3 to *S. lateritius* but do not provide definitive species-level identification. Accordingly, the taxonomic position of MOE3 should be interpreted cautiously until confirmed using higher-resolution molecular approaches [31]. Agarose gel electrophoresis showed that the amplification was effective and produced a distinct PCR product of about 1500 bp, suggesting that the isolate was suitable for molecular characterization [32]. The amplified fragment’s sequencing allowed for precise matching with closely related actinomycete sequences found in public databases and produced trustworthy data for comparative phylogenetic analysis [33]. The BLAST analysis of the partial 16 S rRNA gene sequence showed 99% sequence similarity with members of the genus *Streptomyces*, confirming the isolate MOE3’s membership in the taxonomically and biotechnologically relevant genus. More precisely, the sequence was added to GenBank under the accession number PX944983 as *Streptomyces lateritius* MOE3, and the phylogenetic tree showed that it was closely related to reference strains of *S. lateritius.* This clustering pattern supports the provisional taxonomic placement of isolate MOE3 in close association with reference strains of *S. lateritius* and is consistent with the known diversity of marine-derived Streptomyces species as valuable producers of secondary metabolites [34, 35]. Because species of this genus are well known for their ecological adaptability and ability to synthesize a variety of bioactive chemicals, **t**he provisional assignment of MOE3 to *S. lateritius* is especially noteworthy. As a result, the molecular characterization supported the provisional taxonomic placement of isolate MOE3 and reinforced its potential relevance as a marine actinomycete for future pharmacological and biotechnological investigations. Although partial 16 S rRNA gene sequencing and phylogenetic analysis placed isolate MOE3 in close association with reference strains of *Streptomyces lateritius*, the species-level taxonomic assignment should be interpreted with caution. The 16 S rRNA gene is highly conserved within the genus *Streptomyces*, and closely related species may exhibit very high sequence similarity, thereby limiting the discriminatory power of this marker for definitive species identification. Higher-resolution approaches, including multilocus sequence analysis (MLSA) using conserved housekeeping genes and whole-genome-based analyses, are more appropriate for resolving closely related *Streptomyces* taxa. In particular, genome-wide parameters such as average nucleotide identity (ANI) and digital DNA–DNA hybridization (dDDH) can provide stronger evidence for species-level delineation. These analyses were not available in the present study and therefore represent a limitation of the taxonomic characterization of strain MOE3. Accordingly, the assignment of MOE3 to *S. lateritius* is considered provisional and based on the available 16 S rRNA gene sequence and phylogenetic evidence. Future whole-genome sequencing, followed by ANI, dDDH, and/or MLSA analyses, will be required to confirm its precise taxonomic position.

A comparison with previous Streptomyces studies further clarifies the specific contribution of isolate MOE3. Wibowo et al. [12] described melanin production by the marine-associated *S. cavourensis* SV21, with particular emphasis on the separation and physicochemical characterization of particulate insoluble and water-soluble melanin forms. In contrast, the present study focuses on a mangrove sediment-derived isolate MOE3 from the Egyptian Red Sea, provisionally assigned to *S. lateritius*, and combines pigment production with sequential statistical optimization and biological assessment. El-Zawawy et al. [13] employed Plackett–Burman and Box–Behnken designs for *S. djakartensis* NSS-3 and achieved a 4.19-fold improvement in melanin production; however, this strain originated from terrestrial soil, and its anticancer activity was evaluated against HCT116, HEPG, and MCF7 cell lines. Therefore, the statistical optimization strategy itself is not claimed as novel in the present study. Of particular taxonomic relevance, the marine-derived *Streptomyces* sp. UTMC 1334 showed close relatedness to *S. lateritius* and was investigated for pyrrole derivatives with anti-acetylcholinesterase activity [14], while melanin production was not the focus of that study. More recently, *Streptomyces* sp. SEA5 from Persian Gulf sediment was characterized as a marine melanin producer, with emphasis on pigment structure and antioxidant-related applications [11]. It should also be noted that melanin from *S. glaucescens* NEAE-H has previously been evaluated against the A-431 cell line [9, 36]; therefore, the use of A-431 itself is not considered a stand-alone novelty of the present work. Collectively, the distinctive contribution of MOE3 lies in the integrated characterization of a Red Sea mangrove-derived isolate, provisionally assigned to *S. lateritius*, as a melanin-like pigment producer, together with strain-specific optimization of pigment biosynthesis and subsequent biological evaluation. The melanin-like nature of the recovered pigment was evaluated using UV–Visible spectroscopy and FTIR analysis. The dark pigment produced by isolate MOE3, provisionally assigned to *S. lateritius*, exhibited strong absorption in the UV region followed by a gradual decrease in absorbance toward the visible region, a spectral behavior commonly reported for microbial melanin pigments [13, 19]. The maximum absorbance at 255 nm was also consistent with previous reports of melanin produced by *Streptomyces* species [13, 19]. FTIR analysis further showed absorption bands consistent with hydroxyl, aliphatic C–H, aromatic/conjugated, amide-related, and C–O/C–N-associated vibrations, broadly consistent with spectral features reported for microbial and *Streptomyces*-derived melanins [13, 19, 37]. Collectively, these spectroscopic findings provide supportive evidence for the classification of the MOE3 pigment as a melanin-like pigment rather than definitive structural identification.

Nevertheless, UV–Visible spectroscopy and FTIR provide supportive physicochemical evidence rather than definitive structural identification of melanin. Owing to the heterogeneous, amorphous, and structurally complex nature of microbial melanins, more advanced analytical techniques, including elemental analysis, solid-state NMR, Raman spectroscopy, electron paramagnetic resonance (EPR), XPS, mass spectrometry-based approaches such as LC–MS/MS or Py–GC/MS, and thermal analyses such as TGA/DSC, would provide additional information regarding elemental composition, molecular structure, radical properties, surface chemistry, degradation behavior, and thermal stability. These analyses were not available in the present study and therefore represent a limitation of the current characterization. Accordingly, the pigment is conservatively referred to throughout the manuscript as a “melanin-like pigment” rather than being assigned a definitive melanin structure. Future studies incorporating complementary structural and spectroscopic techniques will be required to establish its precise chemical composition and structural characteristics.

Research on melanin synthesis from melanogenic bacteria indicates that no universal medium or culture conditions are appropriate for their growth. Moreover, the relative abundance and composition of medium components may differ among isolates [6]. Therefore, the application of statistical optimization designs represents an effective strategy for enhancing melanin-like pigment production by identifying the key factors influencing the process and evaluating their interactive effects. In the present study, a two-step statistical optimization approach was employed. The initial Plackett–Burman screening design was used to explore the relative influence of the investigated variables on pigment production. Based on this exploratory screening, starch, KNO₃, MgSO₄·7 H₂O, and incubation period were carried forward for further evaluation using the Box–Behnken design. Within the Box–Behnken experimental runs, the highest experimentally observed response was 3.9 OD, corresponding to approximately a 2.3-fold increase relative to the basal response of 1.7 OD. These observations indicate that the tested factor combinations were associated with higher pigment responses within the investigated experimental region. The negative predicted R² obtained for the full quadratic model warrants cautious interpretation. Predicted R² is based on the model’s ability to predict observations excluded during model fitting and is therefore more sensitive to individual observations than the conventional R². In the present data set, the full quadratic model contained 14 regression terms in addition to the intercept and was estimated from 27 Box–Behnken runs, leaving a relatively limited number of residual degrees of freedom. In addition, several experimental combinations produced responses close to zero, whereas other combinations produced markedly higher absorbance values, increasing the difficulty of representing the entire response pattern using a single quadratic surface. A small number of observations also exhibited relatively large residual deviations and consequently contributed disproportionately to the PRESS statistic. These factors probably contributed to the negative predicted R². Importantly, the overall regression remained statistically significant, and the lack-of-fit test was not significant; however, these results do not overcome the poor cross-validated predictive performance. Therefore, the present model is interpreted primarily as a local descriptive tool for identifying factor effects and interactions within the investigated experimental region rather than as a model for prediction beyond the studied conditions. Additional experimental runs and independent validation would be desirable to improve the predictive robustness of the optimization model. In a previous study, application of the Plackett–Burman design resulted in a 1.23-fold increase in melanin production by *Hortaea werneckii* AS1 [6]. The Box–Behnken design was subsequently used to further evaluate the levels and interactions of the selected components and to improve melanin productivity. A twelve-run Plackett–Burman experiment was used to identify key parameters affecting melanin production by *Aspergillus fumigatus* AFGRD105. This was followed by a Box–Behnken design for further evaluation of the selected components. Melanin productivity increased twofold as a result of the optimization method [38]. Comparison with recent microbial melanin studies further places the present optimization results in context. El-Zawawy et al. reported that melanin production by *Streptomyces djakartensis* NSS-3 increased from 28.38 mg/10 mL under the initial conditions to 118.73 mg/10 mL following Plackett–Burman and Box–Behnken optimization, corresponding to a 4.19-fold enhancement [13]. More recently, Cimini et al. reported a maximum melanin concentration of 2.45 ± 0.01 g/L from *Streptomyces nigra* MT6 after optimization of medium composition, pH, and temperature [10]. In that study, changing the cultivation medium increased melanin production from 0.56 ± 0.02 to 1.91 ± 0.01 g/L, followed by a further approximately 1.3-fold increase to 2.45 ± 0.01 g/L after physiological optimization. Similarly, the marine-derived *Streptomyces* sp. SEA5 was recently reported to produce 1.50 g/L melanin in an L-tyrosine-containing medium and 1.36 g/L when soybean meal was used as an alternative substrate [11].

Direct comparison of absolute pigment yields with the present study is not possible because MOE3 pigment production was evaluated spectrophotometrically at 410 nm and was not quantified gravimetrically as g/L. After correction of the Box–Behnken interpretation, the highest experimentally observed response in the present study was an absorbance of 3.9, representing an approximately 2.3-fold increase relative to the basal response of 1.7. Therefore, the magnitude of improvement was lower than the 4.19-fold enhancement reported for *S. djakartensis* NSS-3, although differences in strain identity, medium composition, response measurement, experimental design, and culture conditions prevent direct quantitative ranking among studies [13]. From a process-development perspective, the approximately 2.3-fold maximum observed increase in melanin-like pigment production demonstrates the potential of culture-condition optimization to improve pigment biosynthesis by isolate MOE3. Nevertheless, the present study was conducted at laboratory shake-flask scale and was designed primarily to identify the culture variables affecting pigment production rather than to establish an industrial production process. Therefore, the current findings should be regarded as an initial step toward process development rather than evidence of demonstrated industrial feasibility. Several additional factors must be considered before industrial-scale production can be proposed. First, pigment production in the present study was monitored spectrophotometrically by absorbance rather than reported as a gravimetric mass yield (g/L). Consequently, direct comparison of volumetric productivity and process yield with industrial fermentation systems remains limited. Determination of dry pigment yield, volumetric productivity, and product recovery efficiency will be necessary in future scale-up studies. Second, the current downstream procedure involves biomass removal by centrifugation, acid precipitation of the extracellular pigment at pH 2 using HCl, repeated centrifugation, and washing steps. Although this procedure is suitable for laboratory-scale recovery, its translation to larger volumes may increase acid consumption, water demand, separation requirements, processing time, and downstream costs. Alternative and simplified recovery strategies should therefore be evaluated during process development. In addition, fermentation economics were not evaluated in the present work. The contribution of medium components, energy requirements for agitation and temperature control, fermentation duration, downstream recovery, and waste treatment to the overall production cost remains unknown. Scale-up would also require validation in controlled bioreactor systems, where parameters such as oxygen transfer, mixing, pH control, aeration, and process reproducibility may differ substantially from shake-flask conditions. Accordingly, a techno-economic assessment integrating pigment yield (g/L), productivity, raw-material costs, downstream recovery efficiency, energy consumption, and process scalability will be required before the economic and industrial feasibility of MOE3-derived melanin-like pigment production can be established.

The biological evaluation of the extracted melanin-like pigment demonstrated a concentration-dependent cytotoxic effect against A-431 human epidermoid carcinoma cells, with an IC₅₀ value of 45.11 ± 0.85 µg/mL after 24 h of exposure. The progressive reduction in cell viability observed in the MTT assay, together with the morphological alterations detected at higher pigment concentrations, indicates that the pigment affects A-431 cell viability under the tested in vitro conditions. However, these findings should be interpreted as preliminary evidence of cytotoxic activity rather than definitive evidence of anticancer efficacy. The present study evaluated only a single malignant cell line, and cytotoxicity toward non-malignant human cells was not assessed. Consequently, a selectivity index could not be calculated, and it remains unknown whether the observed cytotoxicity is preferentially directed toward malignant cells. Furthermore, the MTT assay provides information on cellular metabolic activity and viability but does not establish the mechanism responsible for the observed cytotoxic effect. The cytotoxic activity observed for the MOE3 melanin-like pigment can also be compared with recent Streptomyces-derived melanins, although differences in cell type, exposure conditions, and pigment preparation should be considered. El-Zawawy et al. reported IC₅₀ values of 43.83, 81.99, and 108.9 µg/mL for purified *S. djakartensis* NSS-3 melanin against HEPG, MCF7, and HCT116 cells, respectively [13]. The IC₅₀ of 45.11 µg/mL obtained against A-431 cells in the present study is therefore numerically close to that reported against HEPG cells, but such values should not be interpreted as evidence of equivalent potency because different cancer-cell models were used. A more recent marine study reported an IC₅₀ of 79.82 µg/mL for SEA5-derived melanin against A375 melanoma cells, while the pigment was non-toxic toward normal human dermal fibroblasts and HaCaT keratinocytes. In contrast, normal-cell toxicity was not evaluated in the present study, and therefore selective anticancer activity cannot yet be established [11]. A similar caution applies to antibacterial comparisons. In the present study, MOE3 cell-free supernatant produced inhibition zones of 16–20 mm against the tested bacterial strains, whereas purified melanin from *S. djakartensis* NSS-3 was evaluated quantitatively and showed MIC values of 6.25 µg/mL against the multidrug-resistant *Pseudomonas aeruginosa* strain PA-09 and 25 µg/mL against *Staphylococcus aureus* SA-04 [13]. Because the present study used agar-diffusion screening of cell-free supernatant rather than MIC determination of purified pigment, these antimicrobial results cannot be directly compared in terms of potency. Nevertheless, they demonstrate that antibacterial activity associated with MOE3 is consistent with the broader biological activity reported for other melanin-producing *Streptomyces* strains and warrants quantitative investigation in future studies.

Mechanistic analyses, including apoptosis assessment, intracellular reactive oxygen species generation, mitochondrial membrane potential, cell-cycle distribution, and caspase activation, were not performed in the present study. Similarly, assays evaluating cell migration or wound healing were not conducted. Therefore, no specific mechanism of cell death or effect on cancer-cell migration can be inferred from the current results. Future studies should include non-malignant human cell models to determine the selectivity index, together with complementary mechanistic assays and additional cancer cell lines, before the therapeutic or anticancer potential of the MOE3 melanin-like pigment can be established. Accordingly, potential mechanisms such as oxidative stress, membrane disruption, mitochondrial dysfunction, apoptosis, or cell-cycle alterations should be regarded as hypothetical in the context of the MOE3 melanin-like pigment until they are directly evaluated using appropriate mechanistic assays. The morphological alterations observed in A-431 cells indicate cellular damage but cannot, by themselves, establish a specific molecular pathway or mode of cell death.

### Strengths of the study

A strength of the present study is its integrated investigation of a marine-derived isolate provisionally assigned to *Streptomyces lateritius*, recovered from mangrove sediment along the Egyptian Red Sea coast, as a producer of a melanin-like pigment. The study integrates the isolation of diverse pigmented marine actinomycetes, screening for antibacterial activity, provisional molecular identification of the selected isolate, pigment extraction and spectroscopic characterization, statistical optimization of pigment production, and preliminary evaluation of the extracted pigment for in vitro cytotoxicity against A-431 cells.

### Limitations of the study

Several limitations of the present study should be acknowledged. First, the species-level assignment of isolate MOE3 to *Streptomyces lateritius* was based on partial 16 S rRNA gene sequencing and phylogenetic analysis and should therefore be considered provisional. Second, pigment production was assessed spectrophotometrically rather than quantitatively determined as dry pigment yield (g/L), and the culture conditions evaluated in the present study were not validated at bioreactor scale. Third, classification of the extracted pigment as melanin-like was based primarily on UV–Visible and FTIR spectroscopy. Although these techniques provide supportive physicochemical evidence, definitive structural characterization would require complementary analytical approaches, including elemental analysis, solid-state NMR, Raman spectroscopy, EPR, XPS, mass spectrometry, and thermal analysis. Fourth, although the Box–Behnken model significantly explained variation within the investigated experimental domain, its negative predicted R² indicates limited cross-validated predictive performance and therefore restricts its use for reliable prediction beyond the tested factor space. Regarding the biological evaluation, cytotoxicity was assessed only against A-431 cancer cells, without a non-malignant human cell line, and no selectivity index could therefore be calculated. In addition, mechanistic assays addressing apoptosis, ROS generation, mitochondrial membrane potential, cell-cycle distribution, caspase activation, and cell migration were not performed. Finally, antibacterial activity was evaluated only through an initial agar-diffusion screening assay, without determination of the minimum inhibitory concentration (MIC) or minimum bactericidal concentration (MBC), and without comparison with a standard antibiotic.

### Relevance to the Sustainable Development Goals (SDGs)

The present study is conceptually aligned with several United Nations Sustainable Development Goals (SDGs). The exploration of a marine-derived microorganism as a source of a natural bioactive pigment is relevant to SDG 14 (Life Below Water) by highlighting the biotechnological value of marine microbial biodiversity, particularly microorganisms associated with Red Sea mangrove ecosystems. The development and optimization of microbial pigment production also relate to SDG 9 (Industry, Innovation and Infrastructure) through the application of biotechnology and statistical process optimization to the development of potentially useful natural products. In addition, microbial production of natural pigments may support the principles of SDG 12 (Responsible Consumption and Production) by providing a potential biological alternative to chemically synthesized pigments, although the environmental and economic sustainability of the present process has not yet been established and will require further life-cycle and techno-economic assessment. Finally, the preliminary biological evaluation of the extracted pigment, particularly its in vitro cytotoxic activity, is broadly relevant to SDG 3 (Good Health and Well-being); however, substantially more biological and safety validation is required before any biomedical or therapeutic relevance can be established.

### Future perspectives

Further pigment characterization using complementary approaches, including TLC, SEM coupled with EDX, NMR, Raman spectroscopy, EPR, elemental analysis, XPS, LC–MS/MS or Py–GC/MS, and TGA/DSC, would provide a more comprehensive assessment of its physicochemical and structural characteristics. Whole-genome sequencing followed by ANI and dDDH analyses, and/or MLSA using conserved housekeeping genes, should be undertaken to confirm the precise taxonomic position of MOE3. In addition, systematic stability studies should evaluate the extracted pigment under different pH values, temperatures, storage periods, and light-exposure conditions.

From a production perspective, future work should quantify dry pigment yield in g/L and volumetric productivity, evaluate pigment recovery efficiency, simplify downstream purification, and validate optimized culture conditions in controlled bioreactor systems. Scale-up studies should also assess oxygen transfer, aeration, mixing, pH control, process reproducibility, medium costs, energy requirements, waste generation, and overall techno-economic feasibility.

Biological investigations should include additional cancer cell lines, appropriate non-malignant human-cell models, and calculation of the selectivity index. Mechanistic studies evaluating apoptosis, intracellular ROS generation, mitochondrial membrane potential, cell-cycle alterations, caspase activation, and cellular migration will be required to determine the pathways underlying the observed cytotoxic effect. Moreover, comprehensive antibacterial characterization should include MIC and MBC determination, appropriate reference antibiotics, and statistically supported comparative analyses. Collectively, these investigations will determine whether the MOE3-derived melanin-like pigment can progress from laboratory-scale characterization toward validated biological and biotechnological applications.

## Conclusion

This study demonstrated the production and statistical optimization of a melanin-like pigment by the marine actinomycete isolate MOE3, provisionally assigned to *Streptomyces lateritius* based on partial 16 S rRNA gene analysis. UV–Visible and FTIR analyses provided supportive evidence for classification of the recovered pigment as melanin-like; however, definitive structural identification remains incomplete and will require complementary advanced analytical techniques.

Statistical optimization improved pigment production within the investigated experimental conditions, while the Box–Behnken model was interpreted primarily as a descriptive and exploratory tool because of its limited cross-validated predictive performance. In addition, the extracted pigment exhibited concentration-dependent in vitro cytotoxicity against A-431 cells, with an IC₅₀ of 45.11 ± 0.85 µg/mL after 24 h. These biological findings should be considered preliminary because they were obtained using a single cancer cell line and did not include non-malignant cell controls, mechanistic validation, or in vivo experiments.

Therefore, the present findings do not establish therapeutic, pharmaceutical, or industrial applicability. Further studies should confirm the taxonomic position of MOE3, complete structural characterization of the pigment, quantify pigment yield and productivity, evaluate process performance at bioreactor scale, assess cytotoxic selectivity using normal human cells and additional cancer models, and perform mechanistic and in vivo validation before any biomedical or pharmaceutical application can be considered. Overall, the present study provides an experimental basis for further investigation of MOE3-derived melanin-like pigment rather than evidence of established clinical or industrial utility.

## Data Availability

The 16 S rRNA gene sequence of Streptomyces lateritius MOE3 has been deposited in the GenBank database under the accession number PX944983. All other data generated or analyzed during this study are included in this published article or are available from the corresponding author upon reasonable request.
